# Pattern formation in a 2-population homogenized neuronal network model

**DOI:** 10.1186/s13408-021-00107-1

**Published:** 2021-06-26

**Authors:** Karina Kolodina, John Wyller, Anna Oleynik, Mads Peter Sørensen

**Affiliations:** 1 Faculty of Science and Technology, Norwegian University of Life Sciences, P.O. Box 5003, N-1432 Ås, Norway; 2 Department of Mathematics, University of Bergen, P.O. Box 7803, N-5020 Bergen, Norway; 3 Department of Applied Mathematics and Computer Science, Technical University of Denmark, DK-2800 Kongens Lyngby, Denmark

**Keywords:** Neural field models, Homogenization theory, Turing type of instability, Pattern formation

## Abstract

We study pattern formation in a 2-population homogenized neural field model of the Hopfield type in one spatial dimension with periodic microstructure. The connectivity functions are periodically modulated in both the synaptic footprint and in the spatial scale. It is shown that the nonlocal synaptic interactions promote a finite band width instability. The stability method relies on a sequence of wave-number dependent invariants of $2\times 2$-stability matrices representing the sequence of Fourier-transformed linearized evolution equations for the perturbation imposed on the homogeneous background. The generic picture of the instability structure consists of a finite set of well-separated gain bands. In the shallow firing rate regime the nonlinear development of the instability is determined by means of the translational invariant model with connectivity kernels replaced with the corresponding period averaged connectivity functions. In the steep firing rate regime the pattern formation process depends sensitively on the spatial localization of the connectivity kernels: For strongly localized kernels this process is determined by the translational invariant model with period averaged connectivity kernels, whereas in the complementary regime of weak and moderate localization requires the homogenized model as a starting point for the analysis. We follow the development of the instability numerically into the nonlinear regime for both steep and shallow firing rate functions when the connectivity kernels are modeled by means of an exponentially decaying function. We also study the pattern forming process numerically as a function of the heterogeneity parameters in four different regimes ranging from the weakly modulated case to the strongly heterogeneous case. For the weakly modulated regime, we observe that stable spatial oscillations are formed in the steep firing rate regime, whereas we get spatiotemporal oscillations in the shallow regime of the firing rate functions.

## Introduction

It is common to investigate large-scale activity of neural tissue by means of nonlocal models. Since the seminal works of Amari [1, 2] and Wilson and Cowan [3, 4] such models have been subject to a vast number of investigations, e.g., [5] and the references therein. 1- and 2-population neural field models have been used to understand spatiotemporal dynamics of the cortex of the brain. Stationary spatially-extended patterns are related to visual hallucinations [6–8], while stationary localized structures (*bumps*) are related to short term memory [9–11]. Traveling waves (fronts, pulses, target waves and spirals) are connected to information processing [12, 13].

The 2-population neural field model of the Hopfield type

1 $$\begin{aligned} \begin{aligned} &\frac{\partial }{\partial t} u_{e} = -u_{e} +\omega _{ee} \otimes P_{e}(u_{e}- \theta _{e})- \omega _{ie}\otimes P_{i}(u_{i}-\theta _{i}), \\ &\tau \frac{\partial }{\partial t}u_{i} = -u_{i} +\omega _{ei} \otimes P_{e}(u_{e}-\theta _{e})- \omega _{ii}\otimes P_{i}(u_{i}- \theta _{i}), \end{aligned} \end{aligned}$$

was proposed by Blomquist *et al*. [14]. Here $\omega _{qp}\otimes P_{q}$ is the convolution of $\omega _{qp}$ and $P_{q}$ ($r,s=e,i$) is defined by

2 $$\begin{aligned} \bigl(\omega _{qp}\otimes P_{q}(u_{q}-\theta _{q}) \bigr) (x,t)\equiv \int _{\mathbb{R}^{N}}\omega _{qp}\bigl(x-x' \bigr)P_{q}\bigl(u_{q}\bigl(x',t\bigr)- \theta _{q}\bigr)\,dx'. \end{aligned}$$

This model describes the interaction between populations of excitatory and inhibitory neurons. $u_{e}$ and $u_{i}$ denote the membrane potentials of excitatory and inhibitory neurons, respectively, at the spatial point *x* and time $t>0$. The functions $\omega _{qp}$ ($q,p = e,i$) evaluated at the difference $x-x'$ measure the connectivity strengths between neurons located at position *x* and $x'$, whereas $P_{q}$ ($q=e,i$) are the firing rate functions. $\theta _{e}$ and $\theta _{i}$ are threshold values for firing of the excitatory and the inhibitory neurons, respectively. Notice here that we allow for the situation where $\theta _{e}\neq \theta _{i}$. The parameter *τ* is the relative inhibition time, i.e., $\tau =\tau _{i}/\tau _{e}$ where $\tau _{e}$ ($\tau _{i}$) is the excitatory (inhibitory) time constant.

In [14] the existence and stability of single bumps with firing rate functions in the Heaviside limit have been studied. In Wyller *et al*. [15] pattern formation of the Turing type within the framework of (1) in one spatial dimension as a function of the steepness of the firing rate function was investigated. In particular, formation of stationary periodic patterns and spatiotemporal oscillations were considered.

However, the modeling framework (1) assumes that the cortical medium is homogeneous and isotropic. Thus, the heterogeneity in the cortical structure is not taken into account. Therefore, this modeling approach represents a simplification of the actual situation. One way to take into account the microstructure of the brain media is by using the so-called *homogenization techniques* [16, 17]. The connection between periodic microstructure of the cortex and nonlocal mean field description has been explored in the works [13, 18–23]. It turns out that the microstructure has an impact on the existence and stability of traveling fronts and pulses, such as slowing down and failure of traveling wave propagation. In homogenization techniques for neural field models it is usually assumed that the connectivity functions are represented as $\omega _{qp}^{\varepsilon }(x)=\omega _{qp}(x, x/\varepsilon )$ and have periodicity in the second variable $y=x/\varepsilon $, where the microstructure of heterogeneity is parameterized by $\varepsilon >0$, see, e.g., [24–26]. Thus, a possible extension of (1) taking this type of heterogeneity into account reads

3 $$\begin{aligned} \begin{aligned} &\frac{\partial }{\partial t}u_{e}^{\varepsilon } = -u_{e}^{ \varepsilon } +\omega _{ee}^{\varepsilon }\otimes P_{e} \bigl(u_{e}^{ \varepsilon }-\theta _{e}\bigr)- \omega _{ie}^{\varepsilon }\otimes P_{i}\bigl(u_{i}^{ \varepsilon }- \theta _{i}\bigr), \\ &\tau \frac{\partial }{\partial t}u_{i}^{\varepsilon } = -u_{i}^{ \varepsilon } +\omega _{ei}^{\varepsilon }\otimes P_{e} \bigl(u_{e}^{ \varepsilon }-\theta _{e}\bigr)- \omega _{ii}^{\varepsilon }\otimes P_{i}\bigl(u_{i}^{ \varepsilon }- \theta _{i}\bigr). \end{aligned} \end{aligned}$$

The two-scale convergence method described in [27–29] has been applied by Svanstedt *et al*. [25] to a one-population neural field model with spatial microstructure. By employing the same arguments as in [25], one can show that (3) two-scale converges weakly as $\varepsilon \to 0$ to the system

4 $$\begin{aligned} \begin{aligned} &\frac{\partial }{\partial t} u_{e} = -u_{e} +\omega _{ee} \otimes \otimes P_{e}(u_{e}-\theta _{e})- \omega _{ie}\otimes \otimes P_{i}(u_{i}- \theta _{i}), \\ &\tau \frac{\partial }{\partial t} u_{i} = -u_{i} +\omega _{ei} \otimes \otimes P_{e}(u_{e}-\theta _{e})- \omega _{ii}\otimes \otimes P_{i}(u_{i}- \theta _{i}) \end{aligned} \end{aligned}$$

of coupled nonlinear integro-differential equations of the Hopfield type. Here $\omega _{qp}\otimes \otimes P_{q}(u_{q}-\theta _{q})$ is the *double convolution* of $\omega _{qp}$ and $P_{q}$ ($q,p = e, i$) is defined by

5 $$\begin{aligned} & \bigl(\omega _{qp}\otimes \otimes P_{q}(u_{q}- \theta _{q}) \bigr) (x,y,t) \\ &\quad\equiv \int _{\mathbb{R}^{N}} \int _{Y}\omega _{qp}\bigl(x-x',y-y' \bigr)P_{q}\bigl(u_{q}\bigl(x',y',t \bigr)- \theta _{q}\bigr)\,dy'\,dx', \end{aligned}$$

where $x\in \mathbb{R}^{N}, y\in Y$, and $t>0$. Here $Y=[0,1]^{N}\subset \mathbb{R}^{N}$ is a period cell in $\mathbb{R}^{N}$. Following [28] we identify *Y*-periodic functions with functions defined on the *N*-torus $\mathbb{T}^{N}=\mathbb{R}^{N}/\mathbb{Z}^{N}$.

In the papers [24–26, 30–32] the existence and stability of 1-bumps and 2-bumps within the framework of a homogenized 1-population neural field model have been studied. Here one considers the periodic microstructure variation in both the synaptic footprint and the spatial scale of the connectivity strength. We notice, however, that most investigations in inhomogeneous media use 1-population models as modeling frameworks, while it is not common with studies of 2-population nonlocal neural field models with inhomogeneities. We are not aware of any studies of the microstructure effects on the pattern formation mechanism within such modeling frameworks either. In Kolodina *et al*. [33] the existence and stability of *y*-independent single bumps in the homogenized 2-population model (4) in one spatial dimension ($N=1$) were investigated, with the firing rate functions modeled by means of the Heaviside function.

This serves as a background for the present paper. Our goal is to explore pattern formation within the framework of the homogenized 2-population model (4) in the 1-dimensional spatial setting. Thus, we study the effect of the periodic microstructure on the pattern forming process in this modeling framework. We proceed in a way analogous to Wyller *et al*. [15] for the pattern formation in the translational invariant model (1). We study this process in a scenario with steep firing rate functions and in a scenario representing a shallow firing rate regime. The nonlinear development of the instability is detailed by means of numerical simulations demonstrating spatially and spatiotemporally periodic patterns. The numerical scheme is realized in MATLAB^©^ with the build-in functions *conv*2 and *ode45*. Just as in Wyller *et al*. [15] for the translational invariant case (1), we detect numerically in the steep firing rate regime and in the weakly heterogeneous case that the final stage of the pattern forming process consists of stable spatial oscillations where the shape of each oscillation matches remarkably well with the shape of the 1-bumps found in Kolodina *et al*. [33]. We conjecture that these oscillations are *y*-independent, 1-bump periodic solutions of (4) in the steep firing rate regime. This type of solutions is a natural extension of the 1-bump periodic solutions defined in Kolodina *et al*. [34] for the 1-population Amari model.

The present investigation complements the papers [14, 15], and [33] as well as the works [25, 30, 31], and [24].

The paper is organized as follows: In Sect. 2 we specify the properties of the input functions of model (4). We also formulate a comparison result between the solutions of (4) and the solutions of (1) with $\omega _{qp}(x)$ replaced with the mean value $\langle \omega _{qp}\rangle (x)=\int _{0}^{1}\omega _{qp}(x,y)\,dy$. This comparison result is expressed in terms of the separation distance between these solutions (Theorem 1). In Sect. 3 we list the input data used in the numerical simulations. Section 4 is devoted to the methodology for the linear stability of constant solutions of the homogenized 2-population model. Section 5 is devoted to a numerical study of the nonlinear stage of the instability, whereas Sect. 6 contains the conclusions and an outlook. In Appendix A we prove Theorem 1. In Appendix B we show that the gain band structure is of the finite bandwidth type and consists of a finite set of well separated gain bands. Moreover, we show that for localized connectivity kernels, the gain band structure can be extracted from the stability analysis for (1) with $\omega _{qp}$ replaced with the mean value $\langle \omega _{qp}\rangle $. In Appendix C we develop the theory for excitation of a gain band through a Turing–Hopf bifurcation.

## Model

Let us first specify the input data for models (3) and (4):

The firing rate functions $P_{q}$, $q=e,i$, which are expressed in terms of a scaling function *S* and parameterized by means of the steepness parameter $\beta _{q}$ satisfy the following properties:

6 $$\begin{aligned} \begin{aligned} &P_{q}(u)=S(\beta _{q} u), \\ &P_{q}(u)\rightarrow H(u) \quad\text{pointwise as } \beta _{q} \rightarrow \infty, \\ &S:\mathbb{R}\rightarrow [0,1],\\ &S'\in BC(\mathbb{R}),\qquad S'(u)\geq 0. \end{aligned} \end{aligned}$$

Here $BC(\mathbb{R})$ is the space of bounded continuous functions, whereas *H* is the Heaviside function. This means that there is $S_{\max }'>0$ such that

7 $$\begin{aligned} \bigl\vert S(u)-S(v) \bigr\vert \leq S_{\max }' \vert u-v \vert \quad\text{for all $u,v\in \mathbb{R}$}. \end{aligned}$$

The connectivity kernels $\omega _{qp}$ are expressed in terms of the scaling function Φ and the synaptic footprint functions $\sigma _{qp}$, $q,sp=e,i$ as

8 $$\begin{aligned} &\omega _{qp}(x,y;\alpha _{qp})= \frac{1}{\sigma _{qp}(y;\alpha _{qp})}\Phi \biggl( \frac{x}{\sigma _{qp}(y;\alpha _{qp})} \biggr), \\ &\sigma _{qp}(y;\alpha _{qp})>0,\qquad s_{qp}\equiv \int _{Y} \sigma _{qp}(y;\alpha _{qp}) \,dy=\sigma _{qp}(y;\alpha _{qp}=0), \\ &\sigma _{qp}(y;\alpha _{qp})\rightarrow s_{qp} \quad\text{as $\alpha _{qp}\rightarrow 0$ uniformly in $y\in Y$} \end{aligned}$$

just as in [25, 30, 31, 33], and [24]. Here $\sigma _{qp}$ and its derivatives up to order *r* are 1-periodic, continuous, and bounded functions in *y*. Here *r* is a natural number, $r\geq 1$, whereas $s_{qp}, q,p=e,i$ play the roles as the averaged synaptic footprints. The parameters $\alpha _{qp}\geq 0$, $q,p=e,i$ are referred to as the *heterogeneity parameters*. Notice that the translational invariant case is recovered when $\alpha _{qp}=0$. The connectivity kernels are thus assumed to be periodically modulated in both the spatial scales and the synaptic footprints.

The scaling function Φ is assumed to satisfy the following conditions:

9 $$\begin{aligned} \Phi (\xi )=\Phi (-\xi ),\qquad \Phi (\xi )\geq 0,\qquad \int _{ \mathbb{R}}\Phi (\xi )\,d\xi =1. \end{aligned}$$

Moreover, we also impose the extra condition

10 $$\begin{aligned} \int _{\mathbb{R}}\xi ^{r}\Phi (\xi )\,d\xi < \infty,\quad r \in \mathbb{N}. \end{aligned}$$

Notice the role of the parameter *r*: The parameter *r* measures both the degree of localization of the connectivity kernels $\omega _{qp}$ and the degree of regularity of the synaptic footprint functions $\sigma _{qp}$. Condition (10) will ensure that the growth and decay rate curves detected in Sect. 4 are smooth functions of the wave number. Notice that we will make use of this property in Appendix B (Theorem 2, Theorem 3, Corollary 1, Theorem 4, and Theorem 5) and in Appendix C (Theorem 6).

The normalization condition imposed on the scaling function Φ implies that the connectivity functions are normalized, i.e.,

11 $$\begin{aligned} \int _{\mathbb{R}} \int _{Y}\omega _{qp}(x,y;\alpha _{qp}) \,dy\,dx= \int _{\mathbb{R}}\Phi (\xi )\,d\xi =1. \end{aligned}$$

Following [28], we let $\mathbb{T}=\mathbb{R}/\mathbb{Z}$ denote the 1-torus and identify the 1-periodic functions by those ones which are defined on $\mathbb{T}$. Then introduce the Banach space ${\mathcal{B}}\equiv BC(\mathbb{R}\times \mathbb{T})$ of bounded and continuous functions on $\mathbb{R}\times \mathbb{T}$, equipped with the norm

12 $$\begin{aligned} \Vert f \Vert _{{{\mathcal{B}}}}\equiv \sup_{(x,y) \in \mathbb{R}\times \mathbb{T}} \bigl\vert f(x,y) \bigr\vert . \end{aligned}$$

Now, by proceeding in a way analogous to Potthast *et al*. [35], one can prove that the initial value problem of (4) with the connectivity kernels $\omega _{qp}$ and the firing rate functions $P_{q}$ specified as (6) and (8)–(10), respectively, is globally well-posed in the Banach space ${\mathcal{B}}\times {\mathcal{B}}$ equipped with the norm

13 $$\begin{aligned} \bigl\Vert (f,g) \bigr\Vert _{ {{\mathcal{B}}\times {\mathcal{B}}}} \equiv \Vert f \Vert _{ {{\mathcal{B}}}}+ \Vert g \Vert _{ {{\mathcal{B}}}},\quad (f,g)\in {\mathcal{B}}\times { \mathcal{B}}. \end{aligned}$$

Next, let us summarize the boundedness property of system (4): Introduce $\tau _{q}$ defined by

$$\begin{aligned} \tau _{q}\equiv \textstyle\begin{cases} 1, & q=e, \\ \tau, & q=i, \end{cases}\displaystyle \end{aligned}$$

and let $u_{q}^{(0)}, q=e,i$ denote the components of the solution of (4) with all the nonlocal terms omitted. We readily find that

$$\begin{aligned} u_{q}^{(0)}(x,y,t)=U_{q}(x,y)\exp \biggl[- \frac{t}{\tau _{q}} \biggr],\quad q=e,i, \end{aligned}$$

where $U_{q}\in {\mathcal{B}}, q=e,i$ are the components of the initial condition of (4). Now, by using (6)–(11), we find the uniform bounds

$$\begin{aligned} 0\leq \bigl( \omega _{qp}\otimes \otimes P_{q}(u_{q}- \theta _{q}) \bigr) (x,y,t)\leq 1,\quad (x,y,t)\in \mathbb{R}\times Y\times \mathbb{R}_{0}^{+} \end{aligned}$$

for $q,p=e,i$, from which it follows that each component of the solution of (4) satisfies the comparison property

14 $$\begin{aligned} \bigl\Vert u_{q}-u_{q}^{(0)} \bigr\Vert _{ {{\mathcal{B}}}}(t)\leq 1- \exp \biggl[-\frac{t}{\tau _{q}} \biggr],\quad q=e,i. \end{aligned}$$

Hence we arrive at the following result: If $|U_{q}(x,y)|\leq 1$ for all $(x,y)\in \mathbb{R}\times \mathbb{T}$, then $|u_{q}(x,y,t)|\leq 1$ for all $(x,y,t)\in \mathbb{R}\times \mathbb{T}\times \mathbb{R}_{0}^{+}$. This means that the subset $A=\{u_{q}; |u_{q}|\leq 1\}$ of the phase space is a global attractor for the evolution prescribed by model (4). By appealing to property (14), we also conclude that the nonlinear stage of any instabilities leading to pattern formation will eventually be saturated within the present modeling framework. This property is indeed important to bear in mind in the forthcoming sections of the present paper. Notice that these results are exactly the same as the attractor and boundedness results deduced in the translational invariant case [15]. Last but not least, these results also hold true in the multidimensional situation, i.e., when $N>1$.

We finally compare the solution of model (4) with the solution of the translational invariant model (1) for the case when the connectivity kernels are given by the mean values $\langle \omega _{qp}\rangle $ defined by

15 $$\begin{aligned} \langle \omega _{qp}\rangle (x)= \int _{\mathbb{T}}\omega _{qp}(x,y)\,dy. \end{aligned}$$

Let $U_{\langle \rangle }=(\phi _{e},\phi _{i})$ denote the solution of (1) with the mean values $\langle \omega _{qp}\rangle $ of the connectivity kernels $\omega _{qp}$ in (4) as connectivity kernels and $U=(u_{e},u_{i})$ the solution of (4). Introduce the $L^{1}$-norm of the difference between $\omega _{qp}$ and $\langle \omega _{qp}\rangle $, i.e.,

16 $$\begin{aligned} \Delta K_{qp}&\equiv \bigl\Vert \omega _{qp}-\langle \omega _{qp} \rangle \bigr\Vert _{ {1}} \\ &\equiv \int _{\mathbb{R}} \int _{\mathbb{T}} \bigl\vert \omega _{qp} \bigl(x',y'\bigr)-\langle \omega _{qp}\rangle \bigl(x'\bigr) \bigr\vert \,dx'\,dy',\quad q,p=e,i. \end{aligned}$$

We then define the separation distance $D(t)$ between the solution *U* and $U_{\langle \rangle }$ at the time *t* in terms of norm (13) on the Banach space ${\mathcal{B}}\times {\mathcal{B}}$, i.e.,

17 $$\begin{aligned} D(t)&\equiv \Vert U-U_{\langle \rangle } \Vert _{ {{\mathcal{B}} \times {\mathcal{B}}}}(t) \\ &= \bigl\Vert (u_{e}-\phi _{e},u_{i}-\phi _{i}) \bigr\Vert _{ {{ \mathcal{B}}\times {\mathcal{B}}}}(t)= \bigl[ \Vert u_{e}-\phi _{e} \Vert _{ {{\mathcal{B}}}}+ \Vert u_{i}-\phi _{i} \Vert _{ {{\mathcal{B}}}} \bigr](t). \end{aligned}$$

The following theorem guarantees the continuous dependence of the solutions to (4) on the connectivity kernels:

### Theorem 1

*Let* $T<\infty $ *be a fixed finite time*. *For conditions* (6) *and* (8)*–*(10) *imposed on the firing rate functions and the connectivity kernels*, *respectively*, *we find the bounding inequality*

18 $$\begin{aligned} D(T)\leq M(T)\exp \biggl[ \biggl(1+\frac{1}{\tau } \biggr)\beta _{\max }S_{ \max }'T \biggr] \end{aligned}$$

*for the separation distance between the solution* *U* *and* $U_{\langle \rangle }$ *at the fixed time* *T*. *Here* *M* *is the linear function of* *T* *defined by*

19 $$\begin{aligned} M(T)\equiv D(0)+ \biggl[\Delta K_{ee}+\Delta K_{ie}+ \frac{1}{\tau }( \Delta K_{ei}+\Delta K_{ii}) \biggr]T \end{aligned}$$

*with* $\beta _{\max }\equiv \max \{\beta _{e},\beta _{i}\}$.

The proof of this theorem is relegated to Appendix A. A generalization of Theorem 1 to neural field models expressed in terms of Volterra equations can be found in Burlakov *et al*. [36].

We notice that $\Delta K_{qp}$ is a continuous function of $\alpha _{qp}$ where $\Delta K_{qp}\rightarrow 0^{+}$ as $\alpha _{qp}\rightarrow 0^{+}$. Let us choose the heterogeneity parameters $\alpha _{qp}$ in such a way that $\Delta K_{qp}$ is less than a given tolerance $\delta _{qp}$. For the shallow firing rate regime ($(1+ \frac{1}{\tau })\beta _{\max }S_{\max }\ll 1$), we find by using Theorem 1 that the error in approximation of *U* with $U_{\langle \rangle }$ satisfies the bounding inequality

$$\begin{aligned} D(T)\leq D(0)+ \biggl[\delta _{ee}+\delta _{ie}+ \frac{1}{\tau }(\delta _{ei}+ \delta _{ii}) \biggr]T. \end{aligned}$$

In the regime of steep firing rate functions, we have to take into account the exponential factor on the right-hand side of inequality (18) in the error estimation. In order to keep the error of estimation below a certain threshold, we must reduce $\Delta K_{qp}$ as compared with the shallow firing rate regime. In view of the dependence of $\Delta K_{qp}$ on $\alpha _{qp}$, one way of achieving this is to reduce the values of $\alpha _{qp}$.

## Preliminaries: constant solutions. Input data for the numerical simulations

Just as in the translational invariant case, the constant solutions of (4) satisfy the system

20 $$\begin{aligned} &u_{e}=u_{i}\equiv v_{0}, \\ &F(v_{0})=0, \quad -1< v_{0}< 1, \\ &F(v_{0})\equiv v_{0}+P_{i}(v_{0}- \theta _{i})- P_{e}(v_{0}-\theta _{e}). \end{aligned}$$

In Wyller *et al*. [15] it is shown that equation (20) possesses at least one solution which means that the 2-population model (4) has at least one constant solution. Moreover, the maximal number of constant solutions is five.

In the numerical simulations to be presented, we let the scaling function Φ of the connectivity kernels $\omega _{qp}$ be given as the exponentially decaying function

21 $$\begin{aligned} \Phi (\xi )=\frac{1}{2}\exp \bigl(- \vert \xi \vert \bigr), \end{aligned}$$

whereas the scaling function *S* of the firing rate functions $P_{q}, q=e,i$ is given as

22 $$\begin{aligned} S(u)=\frac{1}{2}\bigl(1+\tanh (u)\bigr). \end{aligned}$$

By (6) and (22) we obtain

23 $$\begin{aligned} P_{q}'\equiv \frac{dP_{q}}{du}(u=v_{0}- \theta _{q})=\frac{1}{2}\beta _{q} \cosh ^{-2}\bigl(\beta _{q}(v_{0}-\theta _{q})\bigr),\quad q=e,i. \end{aligned}$$

Just as in Wyller *et al*. [15] we will make use of $\tau _{H}$ (Hopf-time) and $\tau _{\pm }$ defined by

24 $$\begin{aligned} \tau _{H}\equiv \frac{P_{i}'+1}{P_{e}'-1} \end{aligned}$$

and

25 $$\begin{aligned} \tau _{\pm }\equiv \frac{(\sqrt{F'(v_{0})}\pm \sqrt{P_{i}'P_{e}'})^{2}}{(P_{e}'-1)^{2}}, \qquad F'(v_{0}) \equiv \frac{dF}{du}(u=v_{0})=1+P_{e}'-P_{i}'. \end{aligned}$$

In definition (25) we tacitly assume that $F'(v_{0})>0$. We will clarify the role of $\tau _{H}$ and $\tau _{\pm }$ in Sect. 4. The input parameters used in the forthcoming simulations are given as Set *A* and Set *B* in Table 1. Set *A* represents a scenario with steep firing rate functions, whereas Set *B* gives an example of shallow firing rate functions. Both sets of input parameters guarantee that system (4) has one and only one equilibrium point. Notice also that these sets of input parameters were used in Wyller *et al*. [15].

**Table 1:** The single equilibrium points $U_{0}=(v_{0},v_{0})$ are determined by (20) for the listed parameters $\beta _{q}$ and $\theta _{q}$ ($q=e,i$), $P_{e}'$ and $P_{i}'$ are defined by (23), whereas $\tau _{H}$ and $\tau _{\pm }$ (with $F'(v_{0})>0$) are given by (24) and (25), respectively

Parameters	$\beta _{e}$	$\beta _{i}$	$\theta _{e}$	$\theta _{i}$	$v_{0}$	$P_{e}'$	$P_{i}'$	$\tau _{H}$	$\tau _{-}$	$\tau _{+}$
Set *A*	20	30	0.10	0.12	0.129	7.26	13.94	2.39	1.36	4.20
Set *B*	5	10	0.05	0.10	0.106	2.31	4.98	4.56	1.27	16.35

In the numerical computations we assume that the synaptic footprint functions $\sigma _{qp}$ are given by

26 $$\begin{aligned} \sigma _{qp}(y;\alpha _{qp})=\bigl(1+\alpha _{qp}\cos (2\pi y)\bigr)s_{qp},\quad s_{qp}>0, 0\leq \alpha _{qp}< 1. \end{aligned}$$

Moreover, we fix the averaged synaptic footprints $s_{qp}$ to be given as

27 $$\begin{aligned} s_{ee}=0.35,\qquad s_{ei}=0.48,\qquad s_{ie}=0.60 \quad\text{and}\quad s_{ii}=0.69. \end{aligned}$$

We have chosen four different sets of heterogeneity parameters in the forthcoming numerical computations. These sets are given in Table 2.

**Table 2:** Sets of heterogeneity parameters $\alpha _{qp}$. Set 1 represents the weakly modulated case $0<\alpha _{qp}\ll 1$, Set 2 and Set 3 represent scenarios of the medium heterogeneity parameter, whereas Set 4 is an example on a scenario with strong heterogeneity

Parameters	$\alpha _{ee}$	$\alpha _{ie}$	$\alpha _{ei}$	$\alpha _{ii}$
Set 1	0.01	0.025	0.01	0.025
Set 2	0.35	0.4	0.4	0.35
Set 3	0.6	0.55	0.5	0.65
Set 4	0.9	0.85	0.85	0.9

## Linear stability analysis

In this section we develop the methodology for studying the linear stability of the constant solutions of (4). This analysis is based on the same technique as in Kolodina *et al.* [33]. However, as we consider a different type of solutions and smooth firing rate functions, the operators involved have slightly different expressions, which in turn modify the analysis. Moreover, this analysis presents a generalization of the method presented in Wyller *et al*. [15].

Introduce $U=(u_{e}, u_{i})^{T}$. We conveniently rewrite the homogenized system (4) on the compact form

28 $$\begin{aligned} \mathbf{T}^{-1} \frac{\partial U}{\partial t}= - U + { \mathcal{F}}U,\quad {\mathcal{F}}: {\mathcal{B}}\times {\mathcal{B}}\to {\mathcal{B}} \times {\mathcal{B}} \end{aligned}$$

with

29 $$\begin{aligned} \mathbf{T}= \begin{pmatrix} 1&0 \\ 0&1/\tau \end{pmatrix} \quad\text{and}\quad {\mathcal{F}}U= \begin{pmatrix} {\mathcal{F}}_{ee} u_{e}-{\mathcal{F}}_{ie}u_{i} \\ {\mathcal{F}}_{ei} u_{e}-{\mathcal{F}}_{ii}u_{i} \end{pmatrix}, \end{aligned}$$

where ${\mathcal{F}}_{qp}$, $q,p\in \{e,i\}$ are the Hammerstein operators defined as

30 $$\begin{aligned} ({\mathcal{F}}_{qp}u_{q}) (x,y)= \int _{\mathbb{R}} \int _{\mathbb{T}}\omega _{qp}\bigl(x'-x,y'-y \bigr)P_{q}\bigl(u_{q}\bigl(x',y' \bigr)- \theta _{q}\bigr)\,dy'\,dx'. \end{aligned}$$

Let $U_{0}=(v_{0}, v_{0})^{T}$ denote a constant solution to (28)–(30). Then the linearization of the evolution equation (28) about $U_{0}$ is

31 $$\begin{aligned} \mathbf{T}^{-1}\partial _{t}V=-V+{\mathcal{F}}'_{ {U_{0}}}V, \quad V=(V_{e}, V_{i})^{T}. \end{aligned}$$

Here ${\mathcal{F}}'_{ {U_{0}}}: {\mathcal{B}}\times { \mathcal{B}}\to {\mathcal{B}}\times {\mathcal{B}}$ is the Frechét derivative of the operator ${\mathcal{F}}$ at $U_{0}$

32 $$\begin{aligned} {\mathcal{F}}'_{ {U_{0}}}V= \begin{pmatrix} L_{ee}V_{e}-L_{ie}V_{i} \\ L_{ei}V_{e}-L_{ii}V_{i} \end{pmatrix}, \end{aligned}$$

where

$$\begin{aligned} (L_{qp}V_{q}) (x,y)&=P_{q}'(v_{0}- \theta _{q}) (\omega _{qp}\otimes \otimes V_{q}) \\ &\equiv P_{q}'(v_{0}-\theta _{q}) \int _{\mathbb{R}}\,dx' \int _{\mathbb{T}}\,dy'\omega _{qp} \bigl(x'-x,y'-y\bigr)V_{q} \bigl(x',y'\bigr)\,dx'. \end{aligned}$$

In order to analyze the system of linearized equations (31) further, we must introduce several assumptions on the perturbation

$V(\cdot,\cdot,t)=(V_{e}(\cdot,\cdot,t),V_{i}(\cdot,\cdot,t))^{T}$ at a fixed time *t*. In particular, we assume that the component functions $V_{q}$ $(q=e,i)$ belong to the subspace ${\mathcal{D}}$ of ${\mathcal{B}}$ for which the elements *f* satisfy the following properties:

(i) $\|f\|_{ {1}}(y)< \infty $ and $\|\tilde{f} \|_{ {1}}(y)<\infty $ for all $y \in \mathbb{T}$. Here *f̃* denotes the Fourier transform of *f* with respect to the global coordinate *x*.
(ii) $f(x,\cdot ):\mathbb{T}\rightarrow \mathbb{R}$ is piecewise smooth for all $x\in \mathbb{R}$.

As $\|f\|_{ {1}}(y)\equiv \int _{\mathbb{R}}|f(x,y)|\,dx< \infty $ by assumption, *f̃* exists. The other conditions in (i) guarantee that the function *f* can be reconstructed from *f̃* for all $y\in \mathbb{T}$.

From the properties of $\omega _{qp}$ and $P_{q}$, we conclude that the Frechét derivative ${\mathcal{F}}'_{ {U_{0}}}$ maps ${\mathcal{D}}\times {\mathcal{D}}$ onto ${\mathcal{D}}\times {\mathcal{D}}$. Thus we can compute the eigenvalues of $-\mathbf{T}+ \mathbf{T}{\mathcal{F}}'_{ {U_{0}}}$ by applying the Fourier transformation technique. Let

33 $$\begin{aligned} \begin{aligned}& \tilde{V}_{q}(k,y)= \int _{\mathbb{R}}V_{q}(x,y)e^{-i2\pi xk}\,dx,\qquad V_{q}(x,y)= \int _{\mathbb{R}}\tilde{V}_{q}(k,y) e^{i2\pi xk}\,dk, \\ &\tilde{\omega }_{qp}(k,y)= \int _{\mathbb{R}}\omega _{qp}(x,y)e^{-i2 \pi xk}\,dx,\qquad \omega _{qp}(x,y)= \int _{\mathbb{R}} \tilde{\omega }_{qp}(k,y)e^{i2\pi xk} \,dk. \end{aligned} \end{aligned}$$

In particular, from (8) we conclude that the Fourier-transformations $\tilde{\omega }_{qp}$ exist and are given as

34 $$\begin{aligned} \tilde{\omega }_{qp}(k,y)=\tilde{\Phi }\bigl(k\sigma _{qp}(y)\bigr), \qquad\tilde{\Phi }(k)= \int _{\mathbb{R}}\Phi (\xi )e^{-i2\pi \xi k}\,d \xi. \end{aligned}$$

Moreover, since the connectivity kernels $\omega _{qp}$ for each $y\in \mathbb{T}$ are even and real functions of *x*, $\tilde{\omega }_{qp}$ are even and real functions of *k*.

By the convolution theorem, the Fourier transform of $(-\mathbf{T}+ \mathbf{T}{\mathcal{F}}'_{ {U_{0}}} ) V$ with respect to *x* is given by $(- \mathbf{T}+\mathbf{T}\tilde{{\mathcal{F}}'}_{ {U_{0}}}) \tilde{V}$, where

$$\begin{aligned} &\tilde{{\mathcal{F}}'}_{ {U_{0}}}\tilde{V}= \begin{pmatrix} \tilde{L}_{ee}\tilde{V}_{e}-\tilde{L}_{ie}\tilde{V}_{i} \\ \tilde{L}_{ei}\tilde{V}_{e}-\tilde{L}_{ii}\tilde{V}_{i} \end{pmatrix}, \quad\tilde{V}=( \tilde{V}_{e},\tilde{V}_{i})^{T} \end{aligned}$$

with

$$\begin{aligned} (\tilde{L}_{qp}\tilde{V}_{q}) (k,y)=P_{q}'(v_{0}- \theta _{q}) \int _{\mathbb{T}}\tilde{\omega }_{qp}\bigl(k,y'-y \bigr)\tilde{V}_{q}\bigl(k,y'\bigr)\,dy'. \end{aligned}$$

Next, we introduce the Fourier series

35 $$\begin{aligned} &\tilde{V}_{q}(k,y)=\sum_{n\in \mathbb{Z}} \hat{V}_{q}^{(n)}(k) \exp (i2\pi n y), \end{aligned}$$

36 $$\begin{aligned} &\tilde{\omega }_{qp}(k,y)=\sum_{n\in \mathbb{Z}} \hat{\omega }_{qp}^{(n)}(k)\exp (i2\pi n y), \end{aligned}$$

where

37 $$\begin{aligned} &\hat{V}_{q}^{(n)}(k)= \int _{\mathbb{T}}\tilde{V}_{q}(k,y) \exp (-i2\pi n y) \,dy, \end{aligned}$$

38 $$\begin{aligned} &\hat{\omega }_{qp}^{(n)}(k)= \int _{\mathbb{T}}\tilde{\omega }_{mn}(k,y) \exp (-i2\pi n y) \,dy. \end{aligned}$$

These Fourier-decompositions also exist due to the assumptions imposed on $\omega _{qp}$ and $V_{q}$. Notice that since $\tilde{\omega }_{qp}$ is a real, even in *k*, and 1-periodic function of *y*, the Fourier-coefficients $\hat{\omega }_{qp}^{(n)}(k)$ are real. Moreover, since $\tilde{\omega }_{qp}(k,y)=\tilde{\omega }_{qp}(-k,y)$ for all *y*, we have $\hat{\omega }_{qp}^{(n)}(k)=\hat{\omega }_{qp}^{(n)}(-k)$. Hence $\hat{\omega }_{qp}^{(n)}$ is a well-defined, real-valued function of $\eta =k^{2}$: $\hat{\omega }_{qp}^{(n)}=\hat{\omega }_{qp}^{(n)}(\eta )$. Finally, we have $\hat{\omega }_{qp}^{(n)}(k)=\hat{\omega }_{qp}^{(-n)}(k)$ from which it follows that we can let $n\in \mathbb{N}_{0}=\{0,1,2,3,\ldots\}$ without loss of generality.

Finally, we apply the Fourier transform in the local variable *y* to $(- \mathbf{T}+\mathbf{T}\tilde{{\mathcal{F}}'}_{ {U_{0}}}) \tilde{V}$ and obtain $\mathbf{A}_{n}(\eta )\hat{V}_{n}$, where $\mathbf{A}_{n}(\eta )$, $n\in \mathbb{N}_{0}$, $\eta \in \mathbb{R}_{0}^{+} $ are operators acting on the Fourier coefficients $\hat{V}_{n}=(\hat{V}_{e}^{(n)},\hat{V}_{i}^{(n)})^{T}$. It is easy to see that

$$\begin{aligned} \mathbf{A}_{n}(\eta )\equiv \mathbf{T}\bigl(-\mathbf{I}+ \mathbf{C}_{n}( \eta )\bigr), \end{aligned}$$

where $\{\mathbf{C}_{n}\}_{n\in \mathbb{N}_{0}}$ is the sequence of $2\times 2$-matrices defined by

$$\begin{aligned} \mathbf{C}_{n}(\eta )= \begin{pmatrix} P_{e}'\hat{\omega }_{ee}^{(n)}(\eta )&-P_{i}'\hat{\omega }_{ie}^{(n)}( \eta ) \\ P_{e}'\hat{\omega }_{ei}^{(n)}(\eta )&-P_{i}'\hat{\omega }_{ii}^{(n)}( \eta ) \end{pmatrix}. \end{aligned}$$

Here the parameters $P_{q}'$ ($q=e,i$) are given as

39 $$\begin{aligned} P_{q}'\equiv \frac{dP_{q}}{du}(v_{0}-\theta _{q}), \quad q=e,i. \end{aligned}$$

Simple computation reveals that

40 $$\begin{aligned} \mathbf{A}_{n}(\eta )= \begin{pmatrix} -1+P_{e}'\hat{\omega }_{ee}^{(n)}(\eta )&-P_{i}'\hat{\omega }_{ie}^{(n)}( \eta ) \\ \frac{1}{\tau }P_{e}'\hat{\omega }_{ei}^{(n)}(\eta )&-\frac{1}{\tau }(1+P_{i}' \hat{\omega }_{ii}^{(n)}(\eta )) \end{pmatrix}. \end{aligned}$$

Obviously, the eigenvalues of the operator $-\mathbf{T}+ \mathbf{T}{\mathcal{F}}'_{ {U_{0}}}: { \mathcal{D}}\times {\mathcal{D}}\to {\mathcal{D}}\times {\mathcal{D}}$ are given by the eigenvalues of the matrices $\mathbf{A}_{n}(\eta )$, $n\in \mathbb{N}_{0}$, $\eta \in \mathbb{R}_{0}^{+}$. The linear stability problem thus boils down to a study of

41 $$\begin{aligned} \partial _{t}\hat{V}_{n}=\mathbf{A}_{n}(\eta ) \hat{V}_{n},\quad n \in \mathbb{N}_{0}, \eta =k^{2} \end{aligned}$$

for the Fourier coefficients $\hat{V}_{n}$.

We notice the following property of the stability matrix $\mathbf{A}_{0}\equiv \mathbf{A}_{n=0}$: By replacing $\omega _{qp}$ with the mean value $\langle \omega _{qp}\rangle $ defined by (15) in the translational invariant model (1) and proceeding with the linear stability analysis of the constant solution $U_{0}=(v_{0}, v_{0})^{T}$ as in Wyller *et al*. [15], we end up with $\mathbf{A}_{0}$ given by

42 $$\begin{aligned} \mathbf{A}_{0}(\eta )= \begin{pmatrix} -1+P_{e}'\langle \tilde{\omega }_{ee}\rangle (\eta )&-P_{i}'\langle \tilde{\omega }_{ie}\rangle (\eta ) \\ \frac{1}{\tau }P_{e}'\langle \tilde{\omega }_{ei}\rangle (\eta )&- \frac{1}{\tau }(1+P_{i}'\langle \tilde{\omega }_{ii}\rangle (\eta )) \end{pmatrix} \end{aligned}$$

as the stability matrix for the problem. Here

$$\begin{aligned} \langle \tilde{\omega }_{qp}\rangle (\eta )\equiv \int _{0}^{1} \tilde{\omega }_{qp}(k,y) \,dy,\quad \eta =k^{2}, q,p=e,i \end{aligned}$$

is the mean value of the Fourier transform $\tilde{\omega }_{qp}$ (independent of *y*). A detailed proof of this fact is presented in Appendix B (Theorem 4). This clearly sheds light on the role of $\mathbf{A}_{0}$ in the present stability methodology.

The ODE system (41) can be studied in a way analogous to Wyller *et al*. [15] for the translation invariant case. The eigenvalues $\lambda _{n}^{\pm }$ of the coefficient matrix $\mathbf{A}_{n}$ are expressed in terms of the determinant and trace of the matrix $\mathbf{A}_{n}$, i.e.,

43 $$\begin{aligned} \lambda _{n}^{\pm }(\eta )=\frac{1}{2} \bigl(\varphi _{n}(\eta )\pm \sqrt{\bigl(\varphi _{n}(\eta ) \bigr)^{2}-4\psi _{n}(\eta )} \bigr), \end{aligned}$$

where the sequences of functions $\{\varphi _{n}\}_{n=0}^{\infty }$ and $\{\psi _{n}\}_{n=0}^{\infty }$ are defined as

44 $$\begin{aligned} \varphi _{n}(\eta )&\equiv \bigl(\operatorname {tr}(\mathbf{A}_{n}) \bigr) (\eta )=P_{e}' \hat{\omega }_{ee}^{(n)}( \eta )-1-\frac{1}{\tau }\bigl(1+P_{i}'\hat{\omega }_{ii}^{(n)}( \eta )\bigr), \\ \psi _{n}(\eta )&\equiv \bigl(\det (\mathbf{A}_{n}) \bigr) (\eta ) \\ &=\frac{1}{\tau } \bigl[\bigl(1-P_{e}'\hat{\omega }_{ee}^{(n)}(\eta )\bigr) \bigl(1+P_{i}' \hat{\omega }_{ii}^{(n)}(\eta )\bigr)+P_{e}'P_{i}' \hat{\omega }_{ie}^{(n)}( \eta )\hat{\omega }_{ei}^{(n)}(\eta ) \bigr]. \end{aligned}$$

We introduce the sequence of parameterized curves $\Gamma _{n}:\mathbb{R}_{0}^{+}\rightarrow \mathbb{R}^{2},n\in \mathbb{N}_{0}$ defined as

45 $$\begin{aligned} \Gamma _{n}(\eta )= \bigl(\varphi _{n}(\eta ),\psi _{n}(\eta ) \bigr), \quad\eta =k^{2}\geq 0, \end{aligned}$$

in the invariant plane. Each point on this curve represents a Fourier component in the perturbation imposed on the constant background. Thus, the stability problem boils down to the study of the sequence of composite maps

46 $$\begin{aligned} \eta \overset{\Gamma _{n}}{\longmapsto } \bigl(\varphi _{n}(\eta ),\psi _{n}( \eta ) \bigr)\overset{\lambda _{n}^{\pm }}{\longmapsto } \lambda _{n}^{ \pm }( \eta ), \quad n\in \mathbb{N}_{0}. \end{aligned}$$

Notice that the effect of the microstructure on the linear stability properties is taken care of by a sequence of parameterized curves in the invariant plane and not a single curve as in the translation invariant case. Based on the sequence of composite maps (46), we thus arrive at the following conclusion: $U_{0}$ is stable if all the parameterized curves $\{\Gamma _{n}\}_{n\in \mathbb{N}_{0}}$ remain in the second quadrant of the invariant plane for all *η*, whereas we get instability if at least one curve $\Gamma _{n}$ visits at least one of the other quadrants for some *η*-interval.

Let us investigate the initial points of the curves $\{\Gamma _{n}\}_{n\in \mathbb{N}_{0}}$ defined by (44)–(45). First, we observe that

$$\begin{aligned} \tilde{\omega }_{qp}(0,y)= \int _{\mathbb{R}}\omega _{qp}(x,y)\,dx= \int _{\mathbb{R}}\Phi (\xi )\,d\xi =1. \end{aligned}$$

Hence the Fourier coefficients $\hat{\omega }_{qp}^{(n)}$ at $\eta =0$ are given as

$$\begin{aligned} \hat{\omega }_{qp}^{(n)}(0)=\textstyle\begin{cases} 1, & n=0, \\ 0, & n>0, \end{cases}\displaystyle \end{aligned}$$

from which it follows that the sequence of matrices $\{\mathbf{C}_{n}\}_{n\in \mathbb{N}_{0}}$ evaluated at $\eta =0$ is given by

$$\begin{aligned} \mathbf{C}_{0}(\eta =0)= \begin{pmatrix} P_{e}'&-P_{i}' \\ P_{e}'&-P_{i}' \end{pmatrix} \qquad\text{and}\qquad \mathbf{C}_{n}(\eta =0)= \begin{pmatrix} 0&0 \\ 0&0 \end{pmatrix},\quad n\neq 0. \end{aligned}$$

The corresponding sequence of matrices $\{\mathbf{A}_{n}\}_{n\in \mathbb{N}_{0}}$ evaluated at $\eta =0$ is given as

$$\begin{aligned} \mathbf{A}_{0}(\eta =0)= \begin{pmatrix} -1+P_{e}'&-P_{i}' \\ \frac{1}{\tau }P_{e}'&-\frac{1}{\tau }(1+P_{i}') \end{pmatrix} \quad\text{and}\quad \mathbf{A}_{n}(\eta =0)= \begin{pmatrix} -1&0 \\ 0&-\frac{1}{\tau } \end{pmatrix},\quad n\neq 0. \end{aligned}$$

Notice the role of the matrix $\mathbf{A}_{0}$: The local dynamical counterpart of (28)–(29) is given by

47 $$\begin{aligned} \frac{\partial U}{\partial t}= - \mathbf{T}U + \mathbf{T} { \mathcal{F}}_{0} U, \end{aligned}$$

with

48 $$\begin{aligned} \mathbf{T}= \begin{pmatrix} 1&0 \\ 0&1/\tau \end{pmatrix} \quad\text{and}\quad {\mathcal{F}}_{0} U= \begin{pmatrix} P_{e}(u_{e}-\theta _{e})-P_{i}(u_{i}-\theta _{i}) \\ P_{e}(u_{e}-\theta _{e})-P_{i}(u_{i}-\theta _{i}) \end{pmatrix}. \end{aligned}$$

Thus we conclude that the matrix $\mathbf{A}_{0}$ is the Jacobian of the vector field $-\mathbf{T}U + \mathbf{T}{\mathcal{F}}_{0}U$ evaluated at the equilibrium point $U_{0}=(v_{0},v_{0})$.

We readily find that the initial points of $\{\Gamma _{n}\}_{n\in \mathbb{N}_{0}}$ have the following properties:

49 $$\begin{aligned} \begin{aligned} &\Gamma _{n}(0)= \biggl(-1-\frac{1}{\tau },\frac{1}{\tau } \biggr) \quad\text{for } n>0,\\ &\Gamma _{0}(0)= \biggl(-1+P_{e}'- \frac{1}{\tau }\bigl(1+P_{i}'\bigr), \frac{1}{\tau } \bigl(1+P_{i}'-P_{e}'\bigr) \biggr). \end{aligned} \end{aligned}$$

Hence all the parameterized curves $\{\Gamma _{n}\}_{n>0}$ start in the second quadrant in the invariant plane. Just as in Wyller *et al*. [15], the initial point of the curve $\Gamma _{0}$ is in the second quadrant if

50 $$\begin{aligned} &-1+P_{e}'-\frac{1}{\tau }\bigl(1+P_{i}' \bigr)< 0, \end{aligned}$$

51 $$\begin{aligned} &1+P_{i}'-P_{e}'>0. \end{aligned}$$

According to (25), we have $F'(v_{0})=1+P_{i}'-P_{e}'$, where the function *F* is defined by (20). In the present analysis we assume that $F'(v_{0})>0$ so that condition (51) is fulfilled. For $P_{e}'\leq 1$, condition (50) is satisfied for all $\tau >0$, whereas in the complementary regime $P_{e}'>1$, we must have $0<\tau <\tau _{H}$, where $\tau _{H}$ is defined by (24) in order to ensure (50) to be satisfied. The threshold value $\tau _{H}$ for the relative inhibition time *τ* with the constraint $1< P_{e}'<1+P_{i}'$ yields

$$\begin{aligned} &\varphi _{0}(\eta =0, \tau =\tau _{H})=0, \\ &\frac{d\varphi _{0}}{d\tau }(\eta =0,\tau =\tau _{H})= \frac{1}{\tau _{H}^{2}} \bigl(1+P_{i}'\bigr)>0, \\ &\psi _{0}(\eta =0,\tau =\tau _{H})=\frac{1}{\tau _{H}} \bigl(1+P_{i}'-P_{e}'\bigr)>0 \end{aligned}$$

for the trace function $\varphi _{0}$ and the determinant function $\psi _{0}$ given by (44) with $n=0$. In the corresponding local dynamics this point corresponds to a Hopf bifurcation. Therefore $\tau _{H}$ is referred to as the Hopf point in this local description. For $\tau <\tau _{H}$ with $P_{e}'>1$, the equilibrium point $U_{0}$ is asymptotically stable. Introduce $\tau _{\pm }$ defined by (25). In accordance with [15], $U_{0}$ will in this case be a node if $0\leq \tau \leq \tau _{-}$ or $\tau \geq \tau _{+}$ ($P_{e}' \neq 1$). In the complementary regime it will be a focus within the same local dynamical framework.

Next, let us recover the translational invariant limit of the stability problem. This means that all the heterogeneity parameters are equal to zero, $\alpha _{qp}=0$. In that case

$$\begin{aligned} \mathbf{C}_{n}(\eta )= \begin{pmatrix} 0&0 \\ 0&0 \end{pmatrix},\quad n\neq 0, \end{aligned}$$

and hence $\mathbf{A}_{n}(\eta )=-\mathbf{T}$ for $n\neq 0$. Thus the stability is determined by $\mathbf{A}_{0}$, which in this case is equal to the stability matrix of the translational invariant case, i.e.,

52 $$\begin{aligned} \mathbf{A}_{0}(\eta )= \begin{pmatrix} -1+P_{e}'\tilde{\omega }_{ee}(\eta )&-P_{i}'\tilde{\omega }_{ie}(\eta ) \\ \frac{1}{\tau }P_{e}'\tilde{\omega }_{ei}(\eta )&-\frac{1}{\tau }(1+P_{i}' \tilde{\omega }_{ii}(\eta )) \end{pmatrix}, \end{aligned}$$

where $\tilde{\omega }_{qp}\equiv \tilde{\omega }_{qp}(\eta )$ (independent of *y*). See Theorem 4 in Appendix B.

Let us consider the situation where the heterogeneity parameters $\alpha _{qp}$ satisfy $0<\alpha _{qp}\ll 1$. This case is referred to as *the weakly modulated case*.

The scaling function Φ of the connectivity kernels is absolute integrable. Consequently, the Fourier transform Φ̃ is a uniformly continuous function of its argument, see for example Kolmogorov *et al*. [37]. The assumption that $\sigma _{qp}(y;\alpha _{qp})\rightarrow s_{qp}$ as $\alpha _{qp}\rightarrow 0$ uniformly in $y\in \mathbb{T}$ and the uniform continuity of Φ̃ allow us to interchange the limit operation $\alpha _{qp}\rightarrow 0$ and the integration with respect to *y* in the integrals defining the Fourier coefficients $\hat{\omega }_{qp}^{(n)}$. Hence we get

53 $$\begin{aligned} \hat{\omega }_{qp}^{(n)}(\eta;\alpha _{qp}) \rightarrow \textstyle\begin{cases} \tilde{\omega }_{ei}(\eta ), & n=0, \\ 0, & n\neq 0, \end{cases}\displaystyle \end{aligned}$$

as $\alpha _{qp}\rightarrow 0$ uniformly in *η*. Assume that $n\neq 0$. In this case $\varphi _{n}$ and $\psi _{n}$ defined by (44) satisfy the property $\varphi _{n}(\eta )\rightarrow -1-\tau ^{-1}$ and $\psi _{n}(\eta )\rightarrow \tau ^{-1}$ as $\alpha _{qp}\rightarrow 0$ uniformly in *η*. This means that the sequence of curves $\{\Gamma \}_{n>0}$ contracts to one single point $(-1-\tau ^{-1},\tau ^{-1})$ in the invariant plane in this limit. Hence by the expression for $\lambda _{n}^{\pm }$ given by (43), we find that $\lambda _{n}^{\pm }(k)\rightarrow \frac{1}{2}(-1-\tau ^{-1}\pm |1- \tau ^{-1}|)$ in this limit. We conclude that $\lambda _{n}^{+}(k)\rightarrow \max (1,\tau ^{-1})$ and $\lambda _{n}^{-}(k)\rightarrow \min (1,\tau ^{-1})$ as $\alpha _{qp}\rightarrow 0$ uniformly in *η*. For the case $n=0$, we make use of (43), (44), and (53) to conclude that the eigenvalues $\lambda _{0}^{\pm }(\eta )$ approach the eigenvalues of the matrix $\mathbf{A}_{0}$ given by (52). We conclude that only the $n=0$-mode in the perturbation *V* about the equilibrium $U_{0}$ will have an impact on the linear instability, and hence on the pattern formation when $\alpha _{qp}=0$.

Since $\sigma _{qp}$ by assumption is a continuous function of $\alpha _{qp}$ and the Fourier-transform Φ̃ is a uniformly continuous function of its argument (as a consequence of Φ being absolute integrable), the mapping

$$\begin{aligned} \alpha _{qp}\mapsto \tilde{\Phi }\bigl(k\sigma _{qp}(y; \alpha _{qp})\bigr), \quad\alpha _{qp}\in [0,1) \end{aligned}$$

is a continuous function for each $k\in \mathbb{R}$ and $y\in \mathbb{T}$. The Fourier coefficients $\hat{\omega }_{qp}^{(n)}$ defined by (38) are therefore continuous functions of $\alpha _{qp}$. Hence the trace and the determinant functions $\varphi _{n}$ and $\psi _{n}$ defined by (44) also depend continuously on $\alpha _{qp}$. This continuity property carries over to $\lambda _{n}^{\pm }$ defined by (43). We therefore conclude that the gain band structure in the weakly modulated case emerges as a homotopic deformation of the instability structure detected in the translational invariant case. Thus, we have to search beyond the weakly modulated regime to find significant qualitative changes in the gain band structure as compared with the translational invariant case.

The detailed analysis of the gain band structure is summarized in Theorem 2, Theorem 3, Theorem 4, and Theorem 5 in Appendix B:

1. Theorem 2 implies that all the curves $\{\Gamma _{n}\}_{n>0}$ defined by (45) are closed curves: They start and terminate at the same point $(-1-1/\tau,1/\tau )$ in the second quadrant in the invariant plane. Moreover, the closed curves $\{\Gamma _{n}\}_{n>0}$ contract to the point $(-1-1/\tau,1/\tau )$ as $n\rightarrow \infty $. Theorem 2 also implies that the curve $\Gamma _{0}$ also approaches the point $(-1-1/\tau,1/\tau )$ as $\eta \rightarrow \infty $, but in accordance with (49) it starts at a different point.
2. Theorem 3 implies that the curves $\{\Gamma \}_{n\in \mathbb{N}_{0}}$ remain in the second quadrant for all $\eta \geq 0$ when *n* exceeds a certain threshold, say $n_{0}$. The sequence of curves $\{\Gamma _{n}\}_{0\leq n\leq n_{0}}$ will cross the positive $\psi _{n}$-axis and the negative $\phi _{n}$-axis before returning to the second quadrant in the invariant plane. The latter result simply means that the instability structure is of a finite band width type.
3. By appealing to Theorem 5 we conclude that $\{\Gamma _{n}\}_{n\leq n_{0}}$ forms a sequence of smooth curves in the invariant plane when the scaling function Φ of the connectivity functions $\omega _{qp}$ satisfy the localization property (10) with $r\geq 1$. Moreover, in the generic case these curves exhibit a finite number of transversal crossings with the positive determinant axis and the negative trace axis in this plane. Thus the generic picture of the instability structure consists of a finite set of well-separated smooth gain bands, just as in the translational invariant case.
4. By taking Theorem 3 and Theorem 4 into account, we conclude that the linear stability problem (and consequently also the pattern formation problem) for the homogenized 2-population model can be resolved by using model (1) with the mean values $\langle \omega _{qp}\rangle $ if the scaling function Φ of the connectivity kernels $\omega _{qp}$ satisfies the localization property (10) for the exponent *r* exceeding a threshold value.

Let us illustrate the present analysis with numerical examples where the input data are as given in Sect. 3. We first notice that the Fourier transform Φ̃ of the exponentially decaying scaling function (21) is given by

$$\begin{aligned} \tilde{\Phi }(k)=\frac{1}{(2\pi k)^{2}+1}, \end{aligned}$$

from which it follows that

$$\begin{aligned} \tilde{\omega }_{qp}(k,y)=\tilde{\Phi }\bigl(\sigma _{qp}(y)k\bigr)= \frac{1}{(2\pi \sigma _{qp}(y))^{2}\eta +1}, \quad\eta =k^{2}, \end{aligned}$$

where $\sigma _{qp}(y)$ is given by (26). The corresponding Fourier coefficients $\hat{\omega }_{qp}^{(n)}$ are given by means of the integrals

54 $$\begin{aligned} \hat{\omega }_{qp}^{(n)}(\eta )= \int _{Y} \frac{1}{(2\pi \sigma _{qp}(y))^{2}\eta +1}\exp (-i2\pi n y)\,dy,\quad \eta =k^{2}. \end{aligned}$$

**Figure 1:**
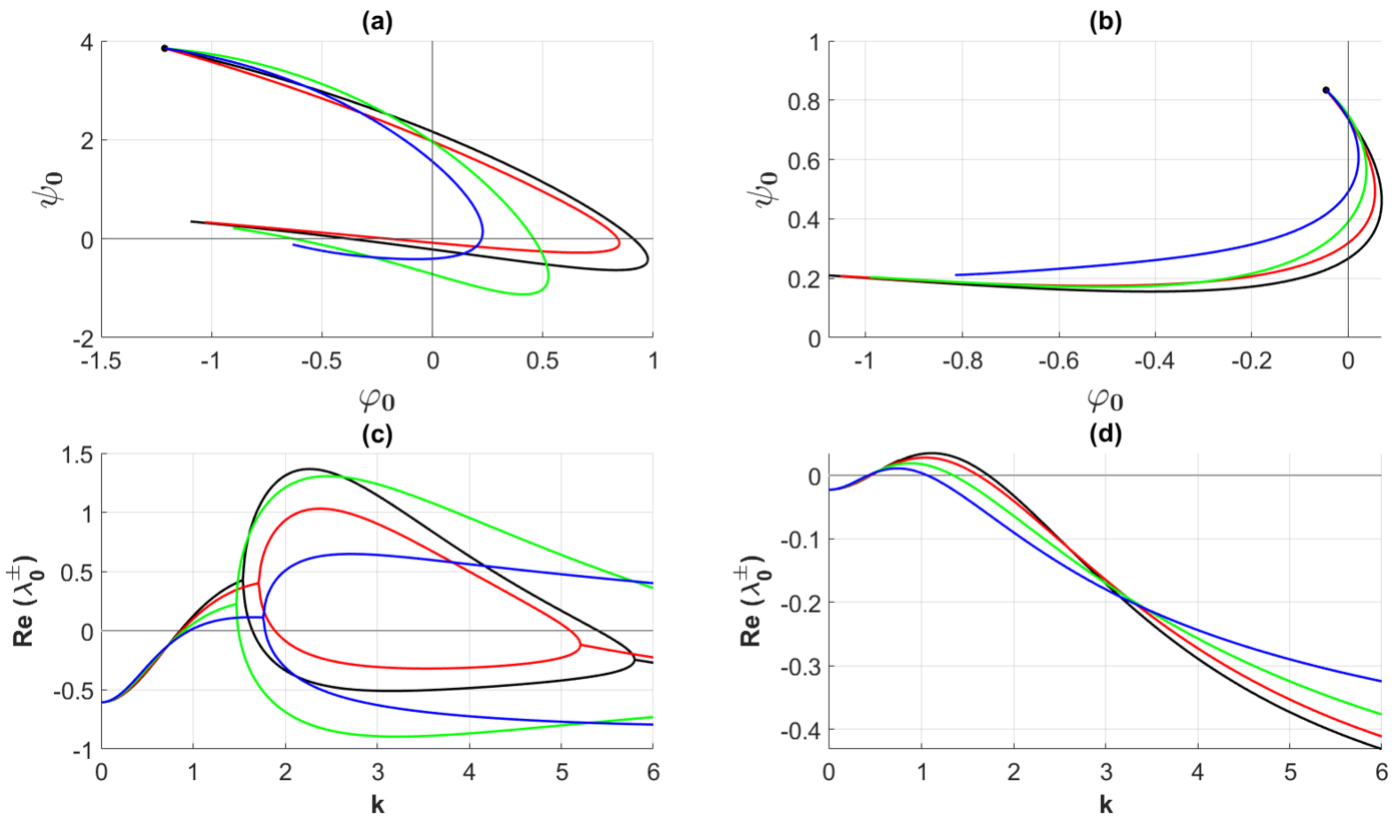
Examples of single gain band structure as a function of the heterogeneity for the Fourier component corresponding to $n=0$. The connectivity functions are given by (8) with (21), the averaged synaptic footprints are fixed as (27). In (**a**) the parameterized curves $\Gamma _{0}$ in the invariant plane defined by means of (44) and (45) are shown for $\tau =2$ and parameter Set *A* (cf. Table 1). In (**c**) the corresponding growth rates ($\operatorname{Re} \{\lambda _{0}^{\pm }\}$ in (43)) are shown as a function of the wave number *k*. In (**b**) the curves $\Gamma _{0}$ are shown for $\tau =4.4$ and parameter Set *B* (cf. Table 1). The corresponding growth rates are shown in (**d**). The black bullet points • in (**a**) and (**b**) are the initial points of the curves $\Gamma _{0}$, $\eta =k^{2}=0$. The heterogeneity parameters producing the black, red, green, and blue curves are Set 1, Set 2, Set 3, and Set 4 in Table 2, respectively

In the forthcoming numerical simulations (i.e., the computations underlying Fig. 1–Fig. 12) we proceed in the same way as in Wyller *et al*. [15] by imposing the bound

55 $$\begin{aligned} \tau _{-}< \tau < \tau _{H}< \tau _{+} \end{aligned}$$

on the relative inhibition time *τ*. Here $\tau _{H}$ and $\tau _{\pm }$ are defined by (24) and (25), respectively. The condition $\tau <\tau _{H}$ guarantees that the initial point of the curve $\Gamma _{0}$ is located in the second quadrant of the trace-determinant plane. According to the analysis of the dynamical system (47)–(48), inequality (55) implies that the equilibrium point $U_{0}=(v_{0},v_{0})$ is an asymptotically stable focus within the framework of this system.

Figure 1 shows the curves $\Gamma _{0}$ in the invariant plane together with the corresponding growth rate curves for the parameter sets in Table 1 and Table 2. For the weakly modulated case ($0<\alpha _{qp}\ll 1$), represented by Set 1 in Table 2, we observe that the curve $\Gamma _{0}$ and the corresponding growth rate curve appear in accordance with (53) as a slight deformation of the corresponding curves produced in the translational invariant case. By adjusting the heterogeneity parameter beyond the weakly modulated case, the following features are notable in the case of steep firing rate functions: Except for Set 3 in Table 2, the maximal growth rate of the instability decreases with the heterogeneity parameter. This property is accompanied by a broadening of the single gain band. In the shallow firing rate regime, we readily observe that the gain band structure is suppressed when increasing the heterogeneity parameter.

**Figure 2:**
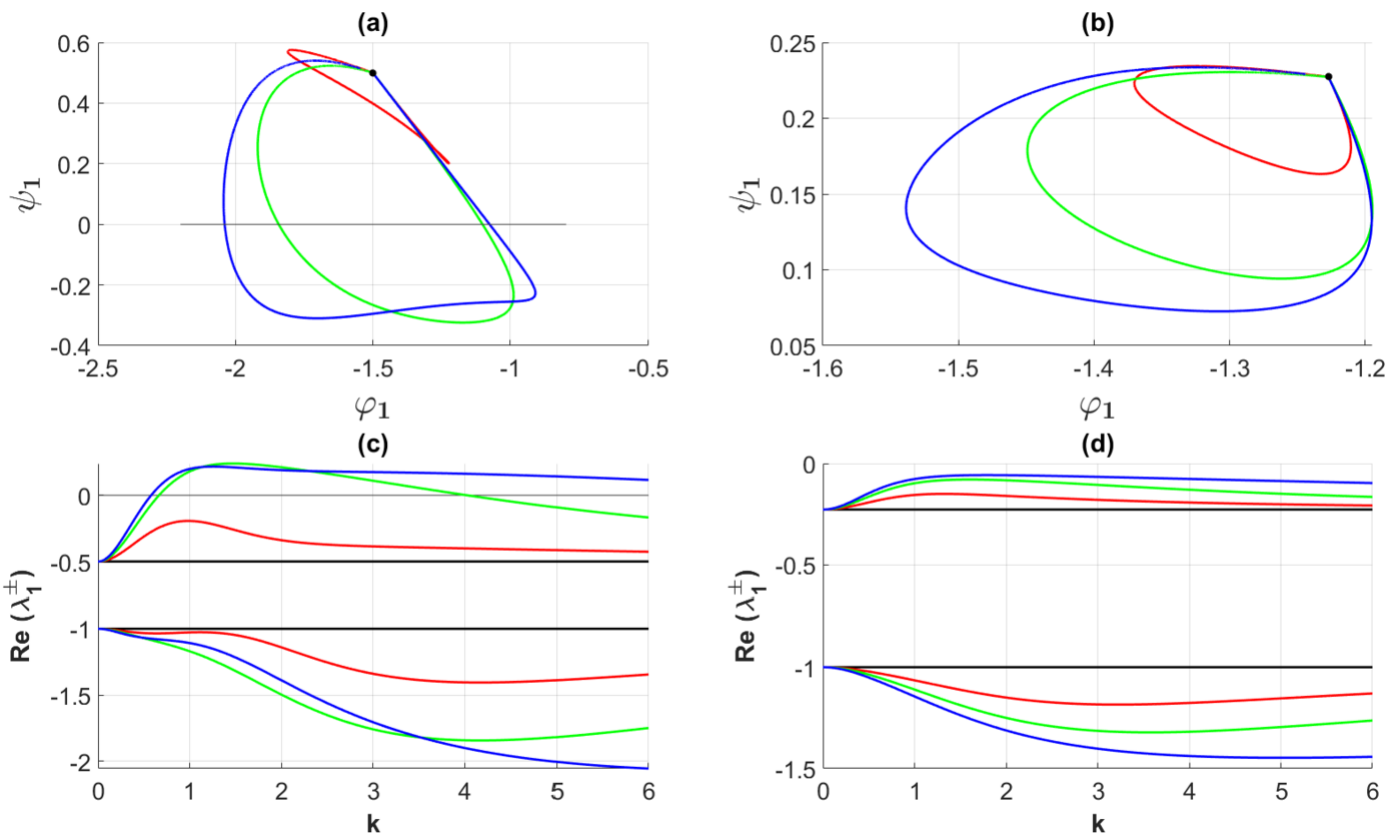
Examples of single gain band structure as a function of the heterogeneity for the Fourier component corresponding to $n=1$. The connectivity functions are given by (8) with (21), the averaged synaptic footprints are fixed as (27). In (**a**) the parameterized curves $\Gamma _{1}$ in the invariant plane defined by means of (44) and (45) are shown for $\tau =2$ and Set *A* (cf. Table 1). In (**c**) the corresponding growth rates ($\operatorname{Re}\{\lambda _{1}^{\pm }\}$ in (43)) are shown as a function of the wave number *k*. In (**b**) the curves $\Gamma _{1}$ are shown for $\tau =4.4$ and Set *B* (cf. Table 1). The corresponding growth rates are shown in (**d**). The black bullet points • in (**a**) and (**b**) are the initial points of the curves $\Gamma _{1}$, $\eta =k^{2}=0$. The heterogeneity parameters producing the red, green, and blue curves are Set 2, Set 3, and Set 4 in Table 2, respectively. Zoomed black curves corresponding to Set 1 are presented in the separate figure, see (**a**) and (**b**) of Fig. 4

In Fig. 2 the curves $\Gamma _{1}$ in the invariant plane are displayed together with the corresponding growth and decay rate curves for the parameter sets in Table 1 and Table 2. We first notice that the curves $\Gamma _{1}$ are closed in agreement with the general theory elaborated in Sect. 4. For the weakly modulated case, represented by Set 1 in Table 2, we show a zoomed version of the parameterized curve in a separate figure (Fig. 4). This figure clearly demonstrates the closed orbit structure of the curve $\Gamma _{1}$ in the vicinity of the point $(-1-1/\tau,1/\tau )$, indicated by the black bullet point •, in the invariant plane, consistent with the analysis presented in Appendix B: The closed curve $\Gamma _{1}$ deforms continuously to the single point • as the heterogeneity parameters tend to zero. Next, for the shallow firing rate regime, the curves are for all the sets of heterogeneity located in total in the second quadrant of the invariant plane, thus showing that the corresponding Fourier component does not contribute to the instability. Interestingly, the curves $\Gamma _{1}$ visit the third quadrant in the invariant plane before returning to the second quadrant in the case of medium-to-strong heterogeneity parameter (represented by Sets 3 and Set 4 in Table 2 in the steep firing rate regime represented by Set *A* in Table 1). In this case the heterogeneity contributes to the linear instability. It is indeed of interest to explore the impact of this instability on the pattern formation process. Here one should notice that it must compete with the contribution from the Fourier mode with $n=0$, and as we will see, it will only have an impact on the initial phase of the pattern forming process and not on the final stage of the pattern forming process.

**Figure 3:**
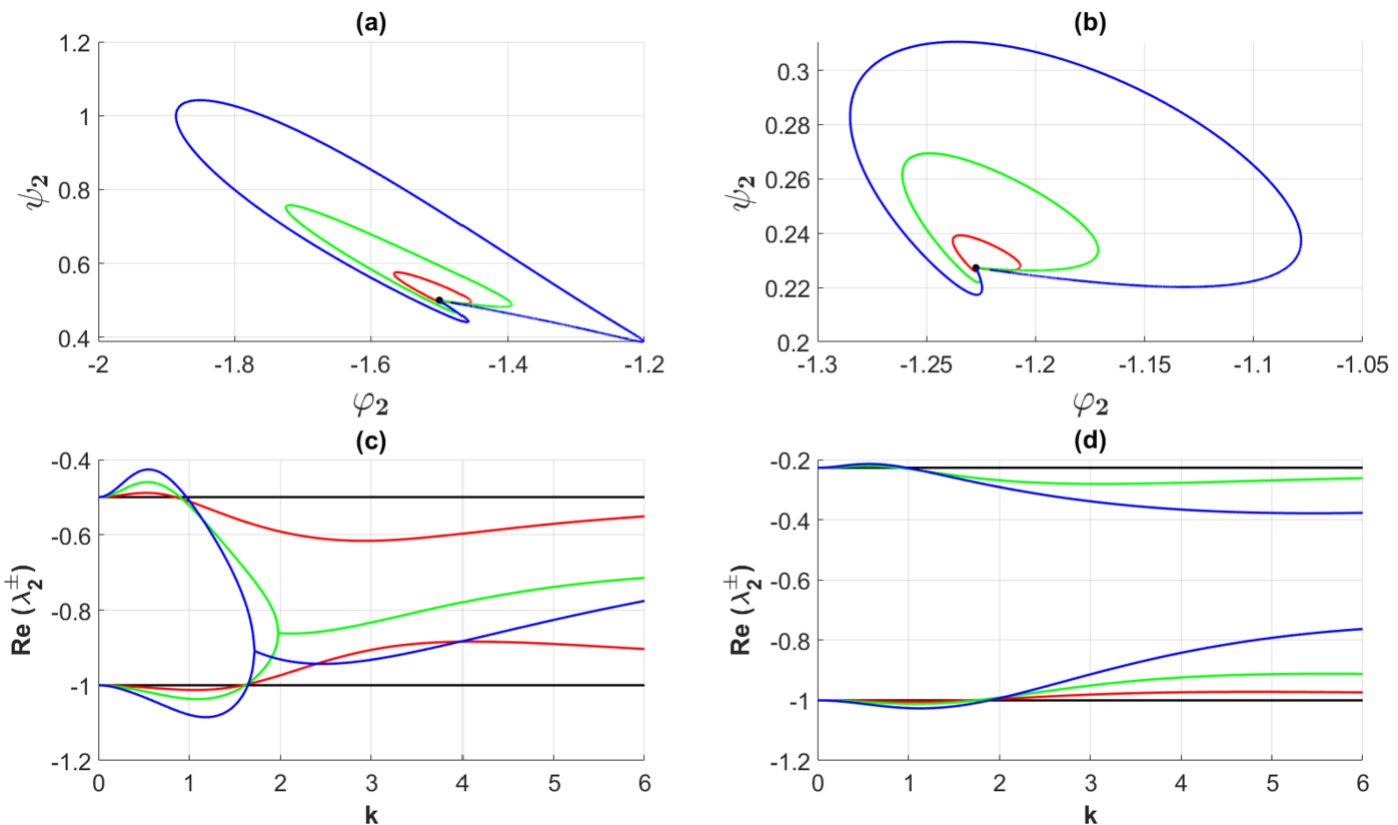
The parameterized curves $\Gamma _{2}$ defined by means of (44) and (45). The connectivity functions are given by (8) with (21), the averaged synaptic footprints are fixed as (27). In (**a**) $\tau =2$ for the firing rate function corresponding to Set *A* (cf. Table 1). In (**b**) the curve $\Gamma _{2}$ is shown for $\tau =4.4$ and Set *B* (cf. Table 1). The black bullet points • in (**a**) and (**b**) are the initial points of the curves $\Gamma _{2}$, $\eta =k^{2}=0$. The heterogeneity parameters producing the black, red, green, and blue curves are Set 1, Set 2, Set 3, and Set 4 in Table 2, respectively. The decay rates corresponding to (**a**) and (**b**) are plotted in (**c**) and (**d**), respectively. Zoomed black curves corresponding to Set 1 are presented in (**c**) and (**d**) of Fig. 4

**Figure 4:**
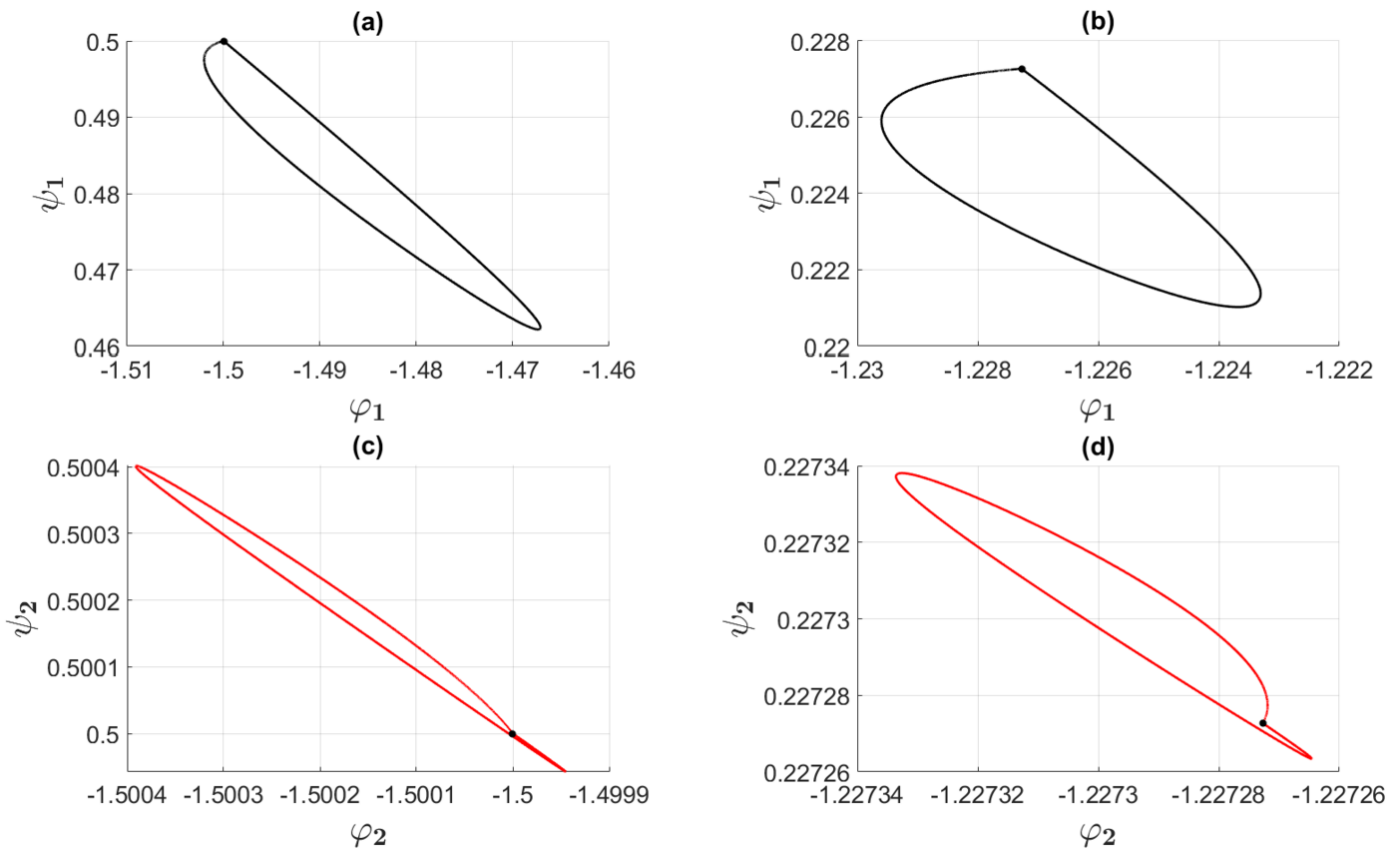
Zoomed versions of the curves $\Gamma _{1}$ and $\Gamma _{2}$ in Fig. 2 and Fig. 3 representing the weakly modulated case, i.e., Set 1 in Table 2, show the closed orbit structure. The connectivity functions are given by (8) with (21), the averaged synaptic footprints are fixed as (27). In (**a**) and (**c**) the parameterized curves $\Gamma _{1}$ and $\Gamma _{2}$ in the invariant plane defined by means of (44) and (45) are shown for $\tau =2$ and Set *A* (cf. Table 1). In (**b**) and (**d**) the curves $\Gamma _{1}$ and $\Gamma _{2}$ are shown for $\tau =4.4$ and Set *B* (cf. Table 1). The black bullet points • in (**a**) and (**b**) are the initial points of the curves $\Gamma _{1}$, $\eta =k^{2}=0$

We have also investigated the parameterized curves $\Gamma _{2}$ given by (44)–(45), see Fig. 3. The outcome of this investigation is summarized in Fig. 3. We find that all these curves are located in the second quadrant of the invariant plane, from which it follows that only the lowest order Fourier components have an impact on the gain band structure, consistent with Theorem 3 in Appendix B. We have depicted separately the parameterized curves $\Gamma _{2}$ for the weakly modulated case, represented by Set 1 in Table 2 in Fig. 4. This figure clearly demonstrates the closed orbit structure of the curve $\Gamma _{2}$ in the vicinity of the bullet point • in the invariant plane, consistent with the previous findings: Just as the curve $\Gamma _{1}$, the closed curve $\Gamma _{2}$ deforms continuously to the single bullet point • as the heterogeneity parameters tend to zero. This is also in agreement with the theoretical results summarized in Appendix B.

We finally detect the generation and coalescence of gain bands by viewing such phenomena as bifurcation processes. Just as in Wyller *et al*. [15], we get two types of bifurcations, a continuous version of the static codimension one bifurcation and a continuous version of the Hopf type of bifurcation. The former one is referred to as the *Turing bifurcation*, whereas the latter one is called the*Turing–Hopf bifurcation*. Paying attention to the fact that we have four heterogeneity parameters which can be varied independently of each other, we can expect a rich plethora of local bifurcation phenomena to take place. In the present study we only give simple examples on these two types of bifurcations. We proceed in the following way: Fix $n\in \mathbb{N}_{0}$ and assemble three of the heterogeneity parameters into a single constant parameter vector which we denote by *â*. The remaining heterogeneity parameter, which is denoted by *α̂*, is varied. We notice that $\hat{a}\in [0,1)^{3}$ and $\hat{\alpha }\in [0,1)$ for case (26). By assumption both $\varphi _{n}$ and $\psi _{n}$ are smooth functions of *α̂*. Moreover, due to (10) (with $r\geq 2$), the same functions are at least two times continuously differentiable with respect to *η*. We will make use of these properties when describing the Turing and the Turing–Hopf bifurcation.

**Figure 5:**
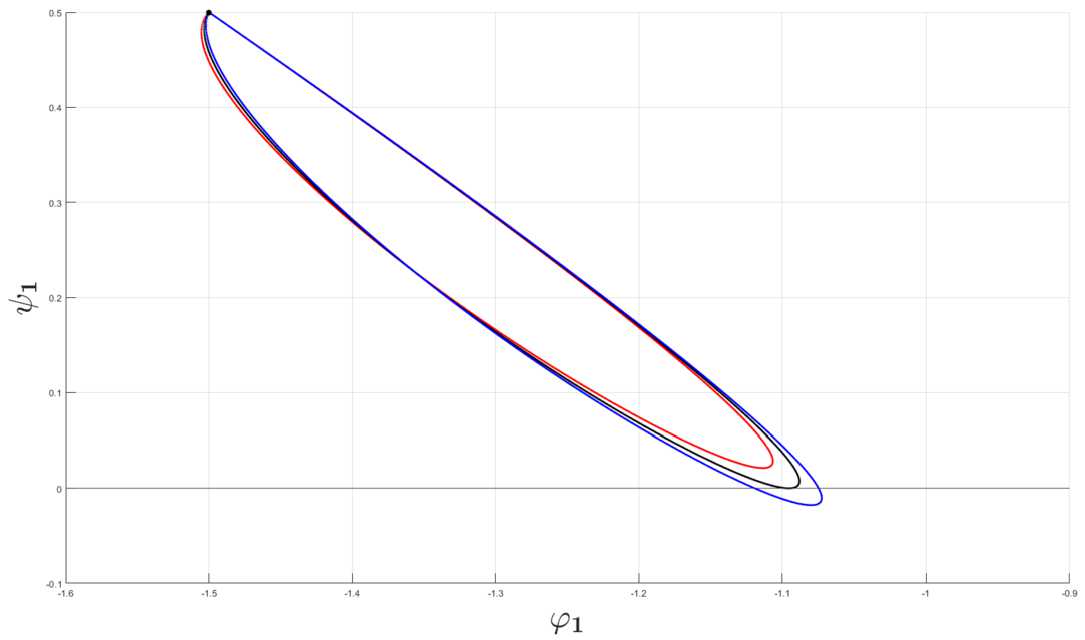
Example of a Turing type of bifurcation (56) for the Fourier component corresponding to $n=1$. The connectivity functions are given by (8) and (21), the averaged synaptic footprints are fixed as (27). The parameterized curves $\Gamma _{1}$ defined by means of (44) and (45) are shown for $\tau =2$ for Set *A* (cf. Table 1). The black point • denotes the initial point of $\Gamma _{1}$. The input heterogeneity parameter vector *â* is given as $\hat{a}=(0.1,0.1,0.1)$ and $\hat{\alpha }=\alpha _{ii}$. The red and the blue curve corresponds to $\alpha _{ii}=0.29$ and $\alpha _{ii}=0.31$, respectively. For $\hat{\alpha }_{c}=0.3009$ (corresponding to the black curve), we have a Turing type of bifurcation
- *Turing–Hopf type bifurcation.* In this case the bifurcation points $(\eta _{c},\tau _{c})$ satisfy the non-transversality condition
  58 $$\begin{aligned} \varphi _{n}(\eta _{c},\tau _{c},\hat{\alpha })=0, \qquad\frac{\partial }{\partial \eta }\varphi _{n}(\eta _{c},\tau _{c}, \hat{\alpha })=0, \quad\hat{\alpha }\in J_{c}. \end{aligned}$$
  Here
  59 $$\begin{aligned} J_{c}=\bigl\{ \hat{\alpha }\in \mathbb{R}_{0};\psi _{n}(\eta _{c},\tau _{c}, \hat{\alpha })>0 \bigr\} . \end{aligned}$$
  We notice that the eigenvalues $\lambda _{n}^{\pm }$ are complex conjugate in the vicinity of the bifurcation points with the real part given as
  $$\begin{aligned} \operatorname{Re}\bigl\{ \lambda _{n}^{\pm }(\eta,\tau,\hat{ \alpha })\bigr\} =\frac{1}{2}\cdot \varphi _{n}(\eta,\tau,\hat{ \alpha }),\quad \hat{\alpha }\in J_{c}. \end{aligned}$$
  We impose the transversality condition
  $$\begin{aligned} \frac{\partial }{\partial \tau }\varphi _{n}(\eta _{c},\tau _{c}, \hat{\alpha })\neq 0,\quad \hat{\alpha }\in J_{c} \end{aligned}$$
  with respect to the control parameter *τ* in order to ensure that the bifurcation point is an isolated point (and not an accumulation point for a sequence of bifurcation points). In this problem the control parameter *τ* depends on the heterogeneity parameters *α̂*. Since the real part of the corresponding eigenvalues $\lambda _{n}^{\pm }$ changes sign at this bifurcation point, the condition
  $$\begin{aligned} \frac{\partial ^{2}}{\partial \eta ^{2}}\varphi _{n}(\eta _{c},\tau _{c}, \hat{\alpha })< 0,\quad \hat{\alpha }\in J_{c} \end{aligned}$$
  means that we have a local maximum of the function $\varphi _{n}$ (or equivalently the real part of $\lambda _{n}^{\pm }$) as a function of *η* at the bifurcation point. When passing through the bifurcation point, a portion of the curve $\Gamma _{n}$ will be contained in the first quadrant in the $\phi _{n},\psi _{n}$-plane for some *η*-interval about $\eta _{c}$. This means that a gain band is excited at the bifurcation point. For the case
  $$\begin{aligned} \frac{\partial ^{2}}{\partial \eta ^{2}}\varphi _{n}(\eta _{c},\tau _{c}, \hat{\alpha })>0, \quad\hat{\alpha }\in J_{c}, \end{aligned}$$
  the function $\varphi _{n}$ (or equivalently the real part of $\lambda _{n}^{\pm }$) has a local minimum as a function of *η* at the bifurcation point. This means that there is an open interval $I_{c}$ about $\eta _{c}$ for which we will have $\varphi _{n}(\eta,\tau,\hat{\alpha })>0$ when $\eta \in I_{c}, \eta \neq \eta _{c}$. This is signaling that a coalescence of gain bands takes place as we pass through the bifurcation point.
  In the translational invariant case the Turing–Hopf bifurcation has been demonstrated numerically in Wyller *et al*. [15], using the relative inhibition time *τ* as a control parameter. The numerical example explored in [15] is based on the exponentially decaying connectivity kernels (i.e., (8) and (26) with Φ given as (21)) with Set *B* in Table 1 as input data. In this case we find by appealing to the implicit function theorem that the bifurcation condition (58) for $n=0$ and $\alpha _{qp}=0; q,p=e,i$ is fulfilled for $\tau =\tau _{c}=4.09$. In Appendix C we prove that this bifurcation is continuously deformed to a Turing–Hopf bifurcation for the weakly modulated case (Theorem 6). We use this result to detect regions in the $\hat{\alpha },\tau $-parameter plane for which $\varphi _{0}(\eta,\tau,\hat{\alpha })>0$ for some *η* and regions for which $\varphi _{0}(\eta,\tau,\hat{\alpha })<0$ for all *η*. The former subset of the $\tau,\hat{\alpha }$-parameter plane corresponds to an excitation of a gain band, whereas as the latter subset produces no such excitation. We have summarized these findings in the color plots in Fig. 6. In the computations underlying these figures, we let the input heterogeneity parameter vector *â* be given as $\hat{a}=0$. Interestingly, Fig. 6 shows that the critical relative inhibition time $\tau _{c}$ for the onset of spatio-temporal oscillations remains almost constant in cases (b) and (c).

- *Turing type bifurcation.* In this case the bifurcation point $(\eta _{c},\hat{\alpha }_{c})$ (where $\eta _{c}=k_{c}^{2}$) is determined by means of the non-transversality condition
  56 $$\begin{aligned} \psi _{n}(\eta _{c},\tau,\hat{\alpha }_{c})=0,\qquad \frac{\partial }{\partial \eta }\psi _{n}(\eta _{c},\tau,\hat{\alpha }_{c})=0,\quad \tau \in \Omega _{c}, \end{aligned}$$
  where
  57 $$\begin{aligned} \Omega _{c}=\bigl\{ \tau \in \mathbb{R}_{+}; \varphi (\eta _{c},\tau, \hat{\alpha }_{c})< 0\bigr\} . \end{aligned}$$
  By using (43), condition (56), and the fact that $\sqrt{\varphi _{n}^{2}}=-\phi _{n}$ (since $\phi _{n}<0$ by assumption, cf. condition (57)), we find that
  $$\begin{aligned} \frac{\partial }{\partial \hat{\alpha }}\lambda _{n}^{+}(\eta _{c}, \tau,\hat{\alpha }_{c})=\varphi _{n}^{-1}(\eta _{c},\tau, \hat{\alpha }_{c})\frac{\partial }{\partial \hat{\alpha }}\psi _{n}( \eta _{c},\tau,\hat{\alpha }_{c}),\quad \tau \in \Omega _{c} \end{aligned}$$
  with respect to the control parameter *α̂*. Hence, in order to ensure that the bifurcation point $(\eta _{c},\hat{\alpha }_{c})$ is isolated (and not an accumulation point for a sequence of bifurcation points), we impose the transversality condition
  $$\begin{aligned} \frac{\partial }{\partial \hat{\alpha }}\psi _{n}(\eta _{c},\tau, \hat{\alpha }_{c})\neq 0,\quad \tau \in \Omega _{c}. \end{aligned}$$
  This means that the corresponding eigenvalue $\lambda _{n}^{+}$ changes sign as we pass the bifurcation point. Notice that due to the conditions imposed on the connectivity kernels make $\frac{\partial }{\partial \hat{\alpha }}\lambda _{n}^{+}(\eta _{c}, \tau,\hat{\alpha }_{c})$ finite. We readily find that
  $$\begin{aligned} \frac{\partial ^{2}}{\partial \eta ^{2}}\lambda _{n}^{+}(\eta _{c}, \tau,\hat{\alpha }_{c})=\varphi _{n}^{-1}(\eta _{c},\tau, \hat{\alpha }_{c})\frac{\partial ^{2}}{\partial \eta ^{2}}\psi _{n}( \eta _{c},\tau,\hat{\alpha }_{c}),\quad \tau \in \Omega _{c}. \end{aligned}$$
  In the process of deriving this result, we have made use of expression (43), the standard rules for differentiation, and thereafter condition (56) (where we take into account that $\sqrt{\varphi _{n}^{2}}=-\phi _{n}$ since $\phi _{n}<0$ by assumption, cf. condition (57)). A notable outcome of this computation is that the terms containing the first and the second derivatives of $\phi _{n}$ with respect to *η* will not be present in the expression for the second derivative of the eigenvalue $\lambda _{n}^{+}$ with respect to *η*.
  This means that $\lambda _{n}^{+}$ has local maximum at the bifurcation point if
  $$\begin{aligned} \frac{\partial ^{2}}{\partial \eta ^{2}}\psi _{n}(\eta _{c},\tau, \hat{\alpha }_{c})>0,\quad \tau \in \Omega _{c}, \end{aligned}$$
  which signals that an excitation of a gain band takes place in this case. $\lambda _{n}^{+}$ has local minimum at the bifurcation point if
  $$\begin{aligned} \frac{\partial ^{2}}{\partial \eta ^{2}}\psi _{n}(\eta _{c},\tau, \hat{\alpha }_{c})< 0,\quad \tau \in \Omega _{c}, \end{aligned}$$
  which means that there is an open *η*-interval about $\eta _{c}$ for which $\lambda _{n}^{+}$ is strictly positive, thus signalling a coalescence of gain bands to take place at the bifurcation point. Figure 2 clearly indicates that a gain band is generated in the steep firing rate function regime when the heterogeneity parameter exceeds a certain threshold. We can analyze the generation mechanism as a Turing type of bifurcation phenomenon, i.e., by means of the non-transversality condition (56). In Fig. 5 we have demonstrated this phenomenon by plotting the closed parameterized curve $\Gamma _{1}$ for three different sets of heterogeneity parameters. Here we have fixed all the heterogeneity parameters except $\alpha _{ii}$, i.e., we let $\hat{\alpha }=\alpha _{ii}$.

**Figure 6:**
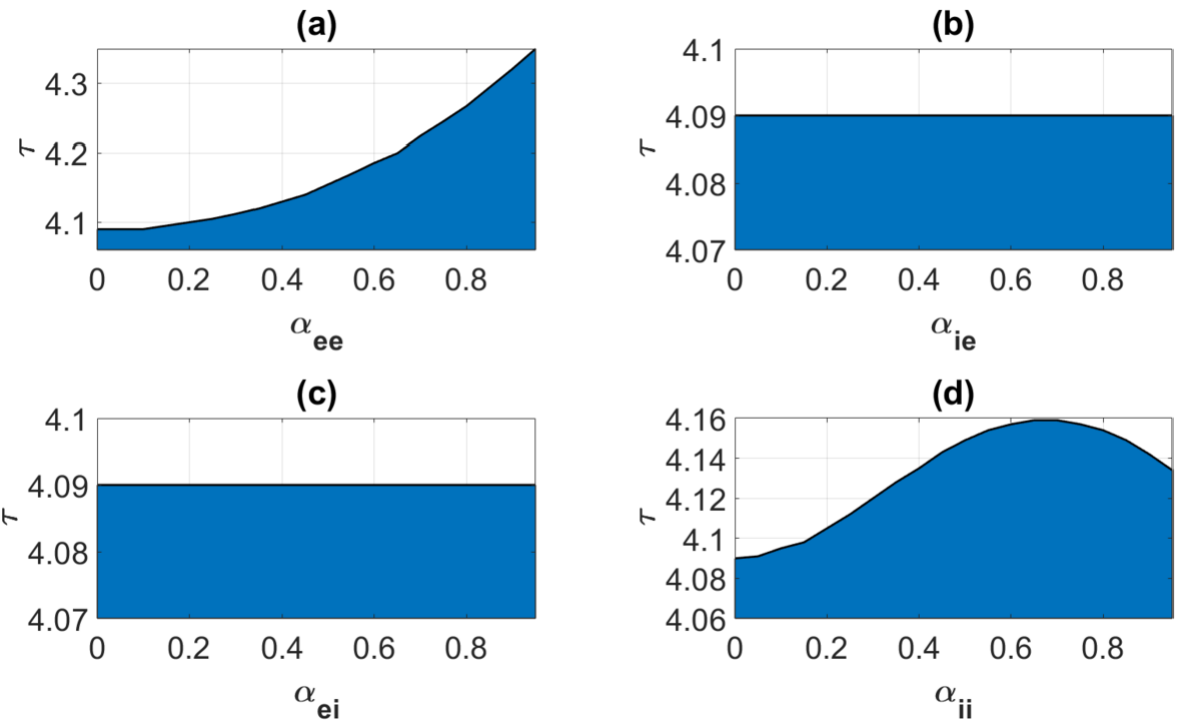
Color plot illustrating the parameter regimes for existence and non-existence of a gain band as a function of the heterogeneity parameter $\alpha _{qp}$; ($q,p=e,i$) and the relative inhibition time *τ*. Blue (transparent) shaded regions correspond to existence (non-existence) of a gain band. The separatrix curve (black) depicts the graph of the critical relative inhibition time $\tau _{c}$ as a function of *α̂*: $\tau _{c}=\tau _{c}(\hat{\alpha }),\hat{\alpha }\in [0,1)$. The connectivity functions are given by (8) and (21) and the synaptic footprint functions as (26) with the averaged synaptic footprints fixed as (27). Input parameters are given by Set *B* in Table 1. (**a**); $q=p=e$, $\hat{a}=(\alpha _{ie},\alpha _{ei},\alpha _{ii})=0$, $\hat{\alpha }=\alpha _{ee}$, in (**b**): $q=i,p=e$, $\hat{a}=(\alpha _{ee},\alpha _{ei},\alpha _{ii})=0$, $\hat{\alpha }=\alpha _{ie}$, in (**c**): $q=e,p=i$, $\hat{a}=(\alpha _{ee},\alpha _{ie},\alpha _{ii})=0$, $\hat{\alpha }=\alpha _{ei}$, and in (**d**): $q=p=i$, $\hat{a}=(\alpha _{ee},\alpha _{ie},\alpha _{ei})=0$, $\hat{\alpha }=\alpha _{ii}$

Notice that the present bifurcation analysis implies that even though we have no bifurcation point for $n=0$ of types (a) and (b), it might happen that at some $n=n_{\ast }$ for which $0< n_{\ast }\leq n_{0}$ we will have a bifurcation point of types (a) and (b). This is caused by the presence of the periodic microstructure in the modeling framework and has no counterpart in the translational invariant limit.

Finally, notice the choice of bifurcation parameters in the Turing and the Turing–Hopf problem: In the Turing–Hopf bifurcation problem (58) we let the relative inhibition time $\tau _{c}$ play the role as the bifurcation parameter. This parameter and $\eta _{c}$) are smooth functions of *α̂*. In the analysis of the Turing bifurcation, it is natural to use the heterogeneity parameter $\hat{\alpha }_{c}$ as the bifurcation parameter. The reason for this is that in accordance with (44) the solution of bifurcation equations (56) is independent of *τ*.

## Pattern formation

Finally, we carry out numerical simulations of (4) in order to illustrate the effect of the periodic microstructure on the pattern forming process. We do this by exploring the nonlinear stage of the instability as a function of the heterogeneity parameter $\alpha _{qp}$. The equations are first discretized in *x* and *y* and then solved using the build-in functions *conv*2 and *ode45* in MATLAB^©^ with the time step $\Delta t=0.1$. In the numerical simulations we consider a finite spatial domain $x\in [-L, L]$ with $L=5$ as an example. We use equidistant grid of the size $N_{x}=201$ in *x*-variables and $N_{y}=11$ in *y*-variables. The convolutions are approximated with the Riemann sums. To avoid an accumulation of errors towards the boundaries of $[-L, L]$, at each time step the convolutions are calculated on $[-2L, 2L]$ in *x* assuming that the solution is 2*L*-periodic in *x*. This assumption on solutions can be justified if the initial conditions are 2*L* periodic as well.

In the numerical simulations to be presented it is assumed that the initial condition for (4) consists of a homogeneous solution $v_{0}$ (given by Table 1) with a small perturbation on the form of a narrow centered rectangular box superimposed, i.e.,

60 $$\begin{aligned} U(x, y)= \textstyle\begin{cases} 0.2, & \text{if } x\in [-0.5, 0.5], y\in [-0.5, 0.5], \\ v_{0}, & \text{if } x\in [-L, L]\setminus [-0.5, 0.5], y\in [-0.5, 0.5]. \end{cases}\displaystyle \end{aligned}$$

We project the numerical solutions onto the $y=1/2$-plane for four different sets of the heterogeneity parameters $\alpha _{qp}$ given in Table 2. Here we divide investigations into two subsections corresponding to steep firing rate regime, i.e., Set *A* in Table 1 (Sect. 5.1), and shallow firing rate regime, i.e., Set *B* in Table 1 (Sect. 5.2).

### Steep firing rate regime (set *A*)

**Figure 7:**
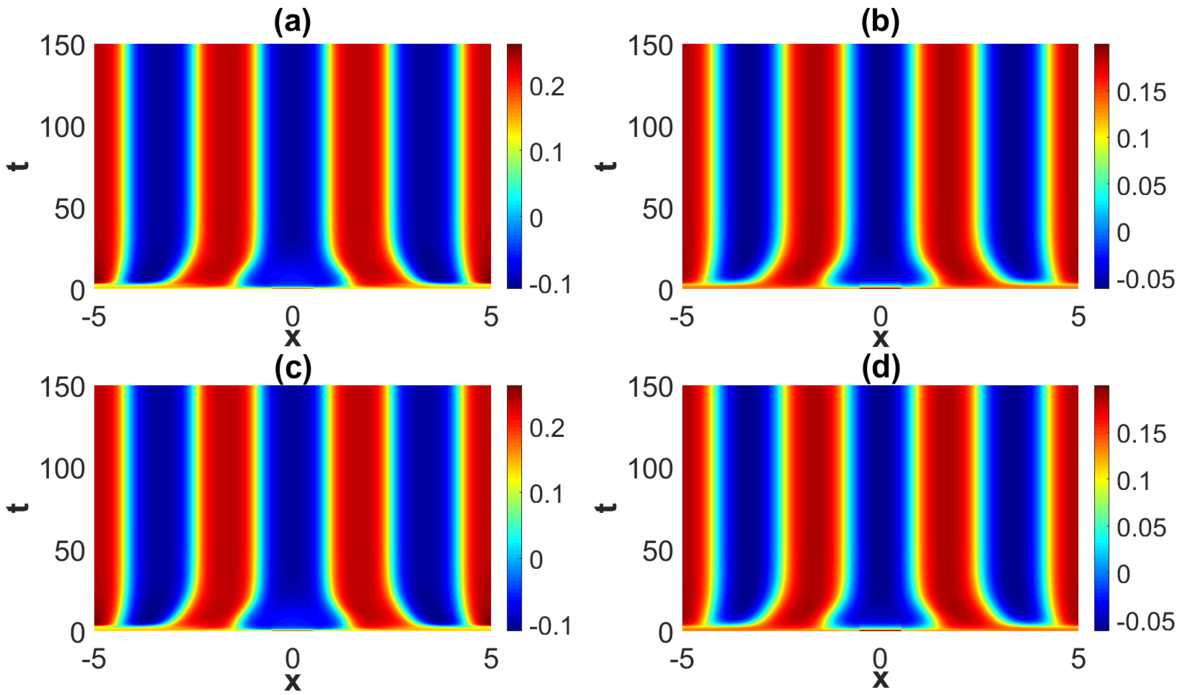
Projection of the numerical solutions of (4) onto the $y=1/2$-plane for parameter Set *A* in Table 1 with $\tau =2$. (**a**) represents the excitatory activity level $u_{e}$ and (**b**) the inhibitory activity level $u_{i}$ in the translational invariant case. (**c**) represents the excitatory activity level $u_{e}$ and (**d**) the inhibitory activity level $u_{i}$ for Set 1 in Table 2. The initial condition is given by (60)

The outcome of the numerical study of the pattern formation in the steep firing rate regime (represented by Set *A*) and in the weakly modulated case (represented by Set 1 in Table 2) is stable *x*-dependent spatial oscillations. See Fig. 7. For the sake of completeness we have compared the outcome in the weakly modulated case with the outcome the translational invariant case, thus confirming the expectation that the dynamical evolution in the weakly modulated case appears as a continuous deformation of the translational invariant case.

**Figure 8:**
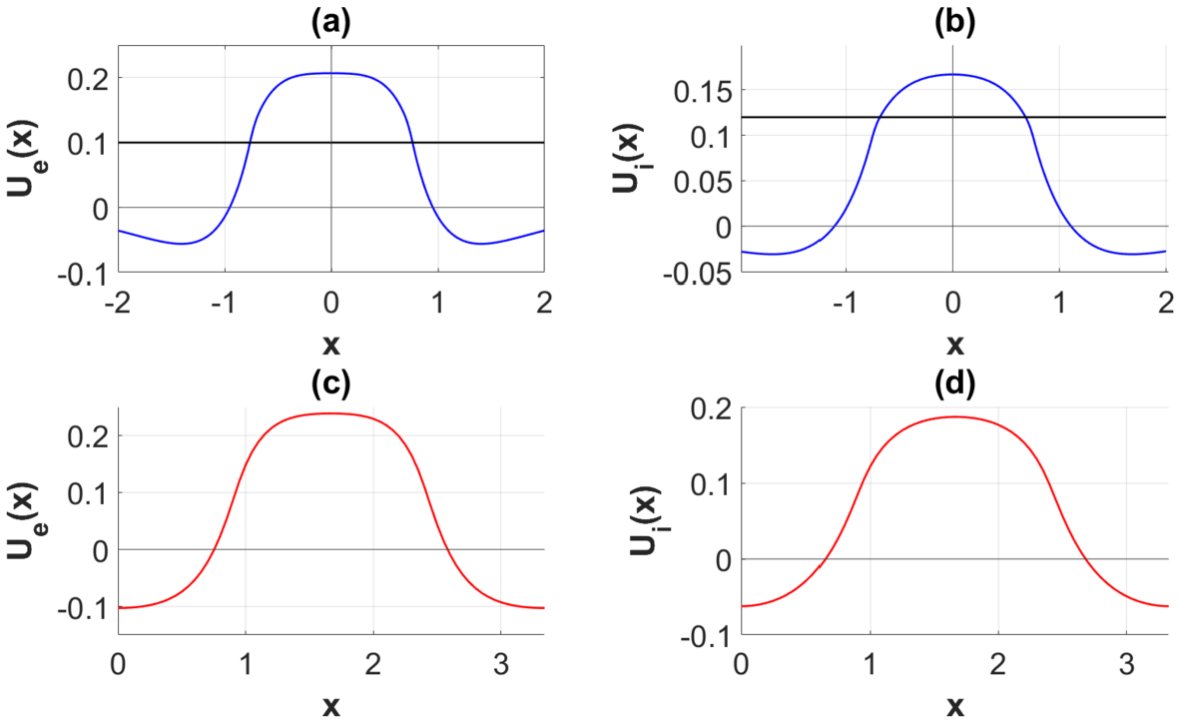
Comparison between stationary broad bump with the Heaviside firing rate function, where (**a**) and (**b**) correspond to the excitatory and the inhibitory components, respectively, with the stationary oscillations (**c**) and (**d**) restricted to one period for Set *A* in Table 1 and Set 1 in Table 2

The shape of each oscillation is remarkably similar to the shape of the 1-bump studied in Kolodina *et al*. [33] for the Heaviside limit of the firing rate functions. This is illustrated by means of the plots in Fig. 8: In (a) and (b) we show the excitatory and the inhibitory component of the stable single bump in the Heaviside limit of the firing rate functions, whereas in (c) and (d) we plot the corresponding components of the spatially oscillating pattern restricted to one period when using heterogeneity parameter Set 1 in Table 2 as input parameters. The wave number of these oscillations is approximately equal to the wave number maximizing $\lambda _{0}^{+}$. The procedure for estimating this wave number goes as follows: The starting point is the sample of wave numbers $S=\{k_{1},{\ldots },k_{N}\}$ underlying the numerical computations leading to the growth rate curves in Fig. 1. The estimate for the wave number which we denote by $k_{\max }$ is selected from the sample *S* in such a way that $\lambda _{0}^{+}(k_{\max })>\lambda _{0}^{+}(k_{i})$ for $k_{i}\in S, k_{\max }\neq k_{i}$. For the input parameters given by Set *A* and Set 1, we find that $k_{\max }\approx 2.31$ in this way. The corresponding wavelength is defined by $\Lambda _{\max }=2\pi /k_{\max }$.

The result summarized in Fig. 8 gives rise to the conjecture that one possible final stage of the pattern forming process in the steep firing rate regime consists of a stable *1-bump periodic solution*. Here we recall what is meant by this notion. The precise definition of 1-bump periodic solutions can be found in Kolodina *et al*. [34] when the modeling framework is the one-population Amari model and the firing rate function is approximated with the Heaviside function. This definition can indeed be extended to our case with a 2-population model: If the firing rate functions are modeled by means of a Heaviside function *H*, the 1-bump $U(x)=(U_{e}(x), U_{i}(x))$ can be defined as a stationary solution of (4) for which the support of $H(U_{q}-\theta _{q}); q=e,i$ (from now on denoted by $\operatorname {supp}(H(U_{q}-\theta _{q}))$) is a compact, connected set, just as in Amari [2] in the one spatial dimension case and in Burlakov *et al*. [38] in the two spatial dimensions case. A 1-bump periodic solution is a stationary periodic solution $U(x)=(U_{e}(x), U_{i}(x))$ to (4) for which the restriction of $\operatorname {supp}(H(U_{q}-\theta _{q})); q=e,i$ on the period interval $[a,a+\hat{T}]$ is a connected, compact set.

Notice that the notions of an *N-bump* and an *N-bump periodic solution* of (4) represent natural extensions of the 1-bump and 1-periodic solution in the Heaviside limit of the firing rate function: $U(x)=(U_{e}(x), U_{i}(x))$ is an N-bump if and only if $\operatorname {supp}(H(U_{q}-\theta _{q})); q=e,i$ is a union of N compact connected disjoint sets, whereas $U(x)=(U_{e}(x), U_{i}(x))$ is an N-bump periodic solution with a period *T̂* if and only if U is *T̂*-periodic and there exists an interval $[a, a+\hat{T}]$ such that $H(U_{q}(a)-\theta _{q}) = 0; q=e,i$ and the restriction of $\operatorname {supp}(H(U_{q}-\theta _{q})); q=e,i $ on $[a, a+\hat{T}]$ is a union of N connected compact disjoint sets. We are not aware of any definition of N-bumps and N-bump periodic solutions for general smooth firing rate functions. However, as the firing rate functions $P_{q}; q=e,i$ are sigmoidal functions which approach the Heaviside function *H* as the steepness parameter $\beta _{q}\rightarrow \infty $, the N-bump and N-bump periodic solutions for steep firing rate functions could be defined as solutions whose limits are their respective counterparts in the Heaviside limit, in a way analogous to Oleynik *et al*. [39], Oleynik *et al*. [40], and Burlakov *et al*. [38]. We do not exclude the possibility that an outcome of the pattern forming process could be structures like N-bump periodic solutions.

**Figure 9:**
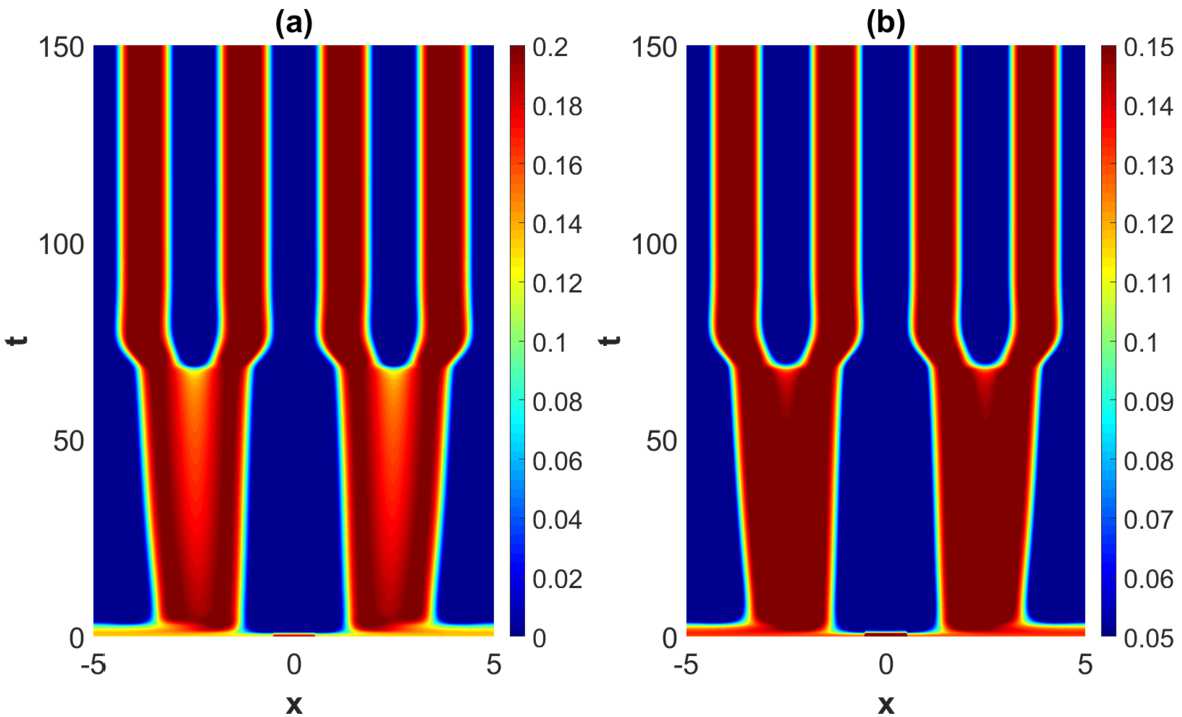
Projection of the numerical solutions of (4) onto the $y=1/2$-plane for parameter Set *A* in Table 1 and Set 2 in Table 2 with $\tau =2$. The projections of the excitatory activity level $u_{e}$ and the inhibitory activity level $u_{i}$ are shown in (**a**) and (**b**), respectively. The initial condition is given by (60)

When increasing the heterogeneity parameters beyond the weakly modulated regime, a rich plethora of phenomena take place. See Fig. 9–Fig. 11. A common and prominent feature in these patterns consists of stabilized spatial oscillatory behavior both in the *x*- and *y*-direction, with the *y*-dependence being as expected more pronounced when increasing the heterogeneity parameter. Again the wavelength in *x*-direction is approximately equal to the wavelength of the linearly most unstable mode, whereas the 1-periodicity in the *y*-direction is retained. The numerical results indicate that the transient phase of the dynamical evolution increases when increasing the heterogeneity parameter to a medium level (Set 2 and Set 3 in Table 2), except for the notable case of strong heterogeneity, represented by Set 4.

Figure 1(c) and Fig. 2(c) show the gain band structure in the steep firing rate regime, i.e., when using Set *A* as input parameters for the computations. Figure 1(c) demonstrates the finite gain band structure for the $n=0$-case, with one unstable band. Interestingly, Fig. 2(a), (c) clearly displays that we get positive growth rates for the linear stage of the $n=1$-instability only for Set 3 and Set 4. Notice that these two sets represent scenarios which model situations well beyond the regimes of weakly modulated case $0<\alpha _{qp}\ll 1$ (represented by Set 1) and the regime of moderate heterogeneity parameter (represented by Set 2). These results are consistent with the comparison result summarized in Theorem 1, the role of the stability matrix $\mathbf{A}_{0}$, and the properties of the gain band structure summarized in Sect. 4: Pattern formation in the regimes of low and moderate heterogeneity parameter is described by means of the translational invariant model with the mean values $\langle \omega _{qp}\rangle $ as connectivity kernels. When comparing the growth rate curves in Fig. 1(c) with the growth rate curve in Fig. 2(c) for the input parameter Set 3 (green curves), we estimate the maximal growth rate for the $n=0$-scenario to be more than six times the maximal growth rate for the $n = 1$-scenario. The comparison for the outcome with Set 4 which models the regime of strong heterogeneity, the maximal growth rate in the $n = 0$-scenario (depicted by the blue curve in Fig. 1(a), (c)) is only three times the maximal growth rate in the corresponding growth rate curve in the $n = 1$-scenario demonstrated in Fig. 2(a), (c). This result indicates that the deviation from the dynamics predicted by the translation invariant model (1) with the mean values $\langle \omega _{qp}\rangle $ as connectivity kernels could eventually show up in the regime of steep firing rate functions-strong heterogeneity. This result is consistent with the comparison result in Theorem 1 and the general properties of the gain band structure summarized in the points 1.–4. in Sect. 4.

Figure 1(b), (d) and Fig. 2(b), (d) show that the shallow firing rate regime corresponding to Set *B* produces a gain band structure only in the $n=0$-case (and not for the $n=1$-case) for Set 1–Set 4 of the heterogeneity parameters. Also this result is consistent with the comparison result stated in Theorem 1 and the role of the stability matrix $\mathbf{A}_{0}$: In this regime the dynamical evolution is basically approximated by means of the translational invariant model (1) with the mean values $\langle \omega _{qp}\rangle $ as connectivity kernels for all values of the heterogeneity parameters $\alpha _{qp}$.

**Figure 10:**
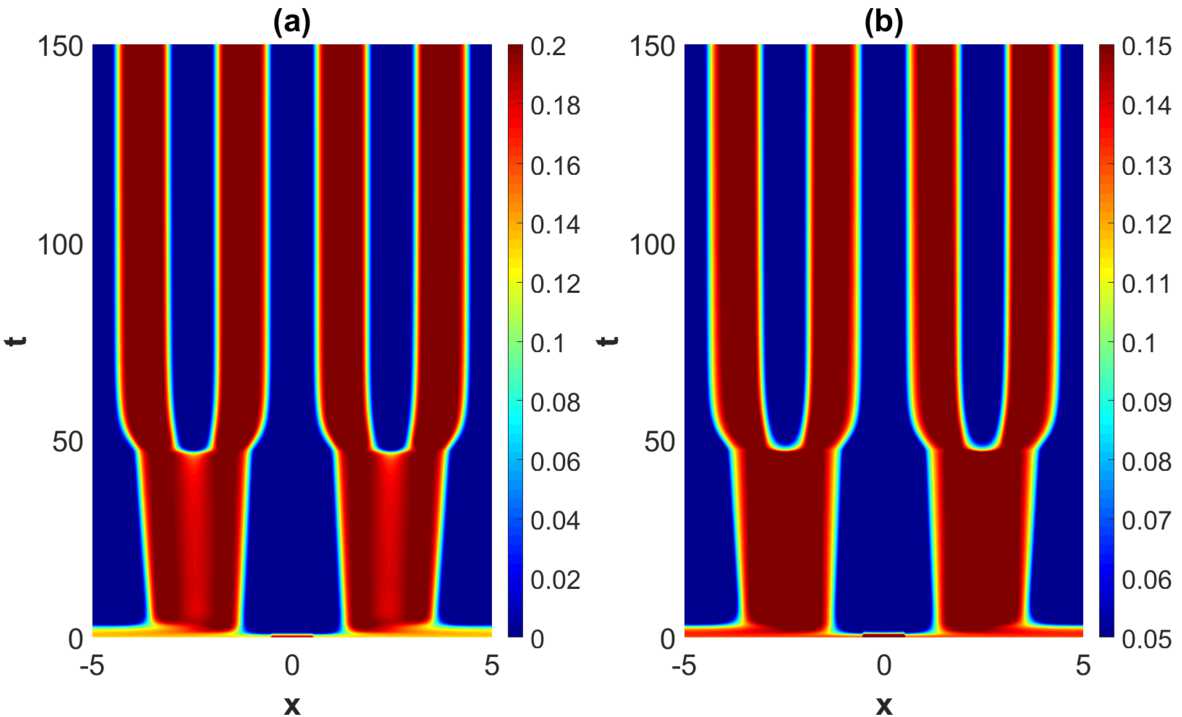
Projection of the numerical solutions of (4) onto the $y=1/2$-plane for parameter Set *A* in Table 1 and Set 3 in Table 2 with $\tau =2$. The projections of the excitatory activity level $u_{e}$ and the inhibitory activity level $u_{i}$ are shown in (**a**) and (**b**), respectively. The initial condition is given by (60)

Interestingly, we numerically detect two characteristic times $t_{1}$ and $t_{2}$ (with $t_{2}>t_{1}$) when using Set 2 and Set 3 in Table 2 as input parameters in the simulations. See Fig. 9 and Fig. 10. The patterns which emerge at the time $t_{1}$ consists of a two-band structure. This structure remains unchanged for the intermediate time interval $(t_{1}, t_{2})$. At the time $t_{2}$ each of these two bands divides into two new bands. The final stage of the pattern forming process thus consists of four stable bands. Notice also that the splitting time $t_{2}$ for the scenario with input data given by Set 2 is greater than $t_{2}$ for Set 3.

**Figure 11:**
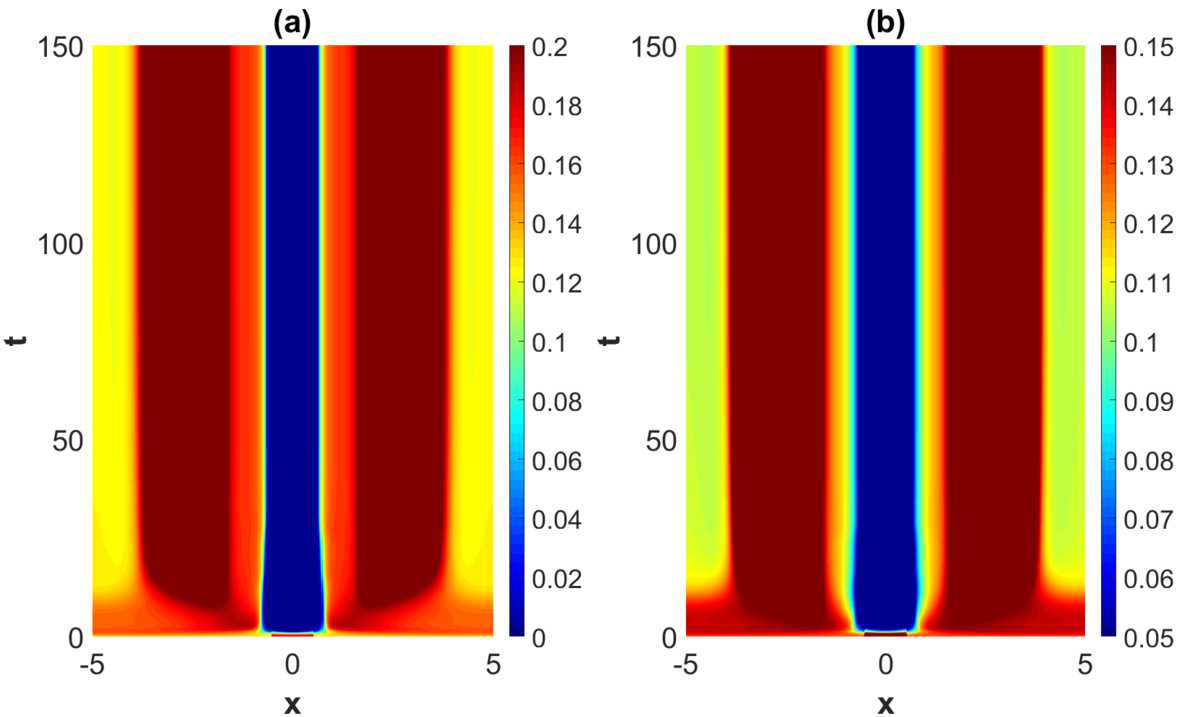
Projection of the numerical solutions of (4) onto the $y=1/2$-plane for parameter Set *A* in Table 1 and Set 4 in Table 2 with $\tau =2$. The projections of the excitatory activity level $u_{e}$ and the inhibitory activity level $u_{i}$ are shown in (**a**) and (**b**), respectively. The initial condition is given by (60)

### Shallow firing rate regime (set *B*)

**Figure 12:**
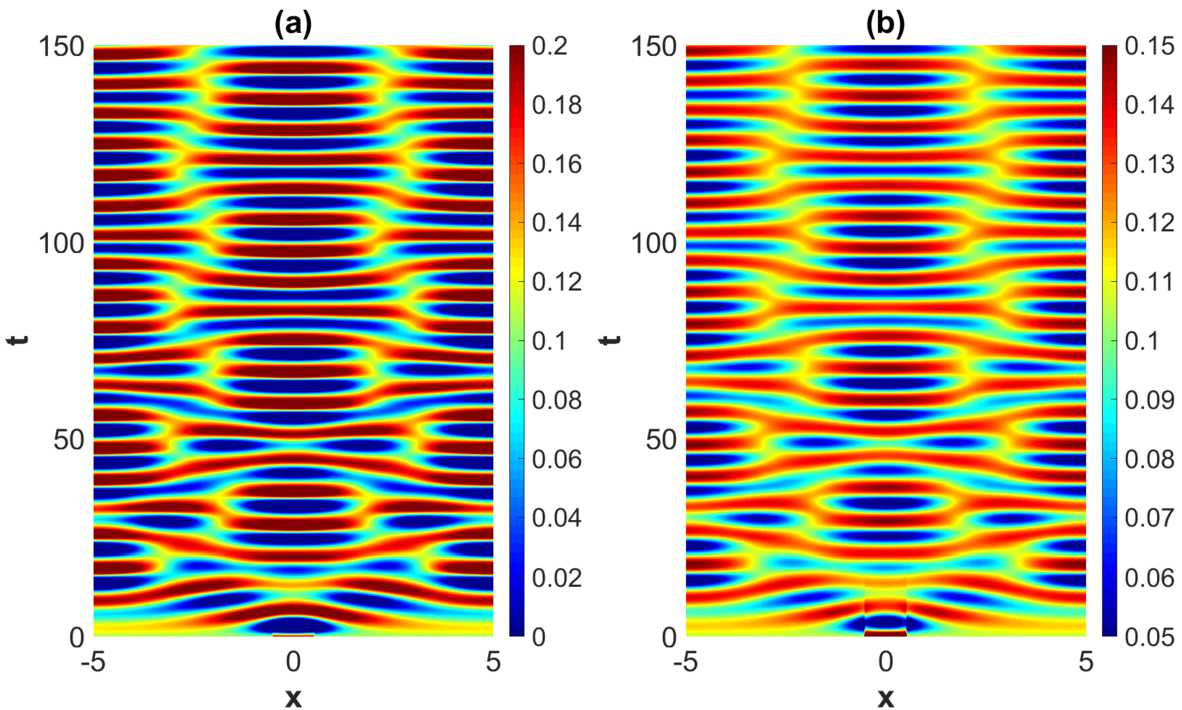
Projection of the numerical solutions of (4) onto the $y=1/2$-plane for parameter Set *B* in Table 1 and Set 3 in Table 2 with $\tau =4.4$. The projections of the excitatory activity level $u_{e}$ and the inhibitory activity level $u_{i}$ are shown in (**a**) and (**b**), respectively. The initial condition is given by (60)

For the shallow firing rate functions case (Set *B* in Table 1) the instability develops into spatiotemporal oscillations. This is consistent with the transient phase described by two complex conjugate eigenvalues of the matrix $\mathbf{A}_{0}$, where the imaginary part determines the frequency of the oscillations. The wavelength of the emerging spatiotemporal oscillations is approximately given by the wavelength corresponding to the maximal growth rate $k_{\max }$, whereas the frequency is given by the imaginary part of the eigenvalues evaluated at $k=k_{\max }$. This is also consistent with the findings in the translational invariant case. Thus, the numerical runs show that the nonlinear stage of the instability in the weakly modulated regime appears as a continuous deformation of the nonlinear stage in the translational invariant case. A notable feature is, however, that the stabilization on spatiotemporal oscillations is slow compared with the emergence of stable spatial oscillations for the steep firing rate case, Set *A*. This is indeed consistent with the findings in the initial stage of the pattern forming process which is described by means of the linearized dynamics as summarized in terms of the gain bands visualized in Fig. 1: The maximal growth rate in the steep firing rate regime turns much greater than the maximal growth in the shallow firing rate regime for the input heterogeneity parameters given by Set 3 in Table 2. This pattern forming process is visualized in Fig. 12 in terms of the numerical solutions projected onto $y=\frac{1}{2}$-plane for the input parameters given by Set *B* in Table 1 and Set 3 in Table 2 with $\tau =4.4$. For the other input parameter sets in Table 2, we will observe the same type of features in the numerical simulations. A notable feature is that the increase in the heterogeneity parameter decreases the growth rate of the fastest growing mode, thus explaining the slowdown of the pattern forming process when increasing the heterogeneity parameter. We finally notice that the outcome of the linear stability analysis for the shallow firing rate case and the simulation results summarized in Fig. 12 is consistent with the predictions of Theorem 1 for shallow firing rate functions.

## Conclusions and outlook

In the present paper we have investigated the effect of periodic microstructure on the pattern formation mechanism in a 2-population neural field model. This work presents an extension of the previous paper by Wyller *et al*. [15] on Turing type of instability and pattern formation within the framework of a 2-population neural field model with homogeneous and isotropic connectivity strengths.

The structure of the linear instability consists of a finite set of well-separated gain bands. In the weakly modulated case the gain band structure emerges as a homotopic deformation of gain band structure in the translational invariant case, due to the continuous dependence of the heterogeneity parameters. We have examples for which gain bands are generated through a Turing type of bifurcation and a Turing–Hopf type of bifurcation. Notice that the instability structure which we have detected for model (4) resembles the gain band structure obtained for modulational instability (MI) in the nonlocal, nonlinear Schrödinger equation [41] and for modulational instability in the nonlocal $\chi ^{(2)}$-model [42]. The existence of several coexisting gain bands thus seems to be a typical feature of nonlocal models.

For the shallow parameter regime, Theorem 1 implies that the dynamical evolution prescribed by the homogenized model can be approximated by the dynamical evolution of the translational invariant case, with the connectivity kernels replaced by means of their respective means values. For the complementary regime of steep firing rate equations, we find that the dynamical evolution depends sensitively on the degree of localization of the scaling function Φ of the connectivity kernels, measured by means of condition (10): An increase in the exponent *r* will lead to stability matrices $\mathbf{A}_{n}$, $n>n_{0}$ where $n_{0}\rightarrow 1$ for which the corresponding $\Gamma _{n}$-curves are totally located in the second quadrant of the trace-determinant plane. See Appendix B. The outcome of the numerical simulations is indeed consistent with this analysis: For the shallow firing rate case, exemplified with Set *B* in Table 1), we find that the gain band structure is completely determined by means of the stability matrix $\mathbf{A}_{0}$, whereas for the steep case illustrated by Set *B* in Table 1 the stability matrix $\mathbf{A}_{1}$ contributes to the gain band structure, in addition to $\mathbf{A}_{0}$.

The development of the linear instability into the nonlinear regime has been detailed numerically. We have considered examples with steep and shallow firing rate functions. In order to compare with previously obtained results for the 2-population neural translational invariant model, we have projected the numerical solutions onto the $y=\frac{1}{2}$-plane and for the same parameter sets for the steep and shallow regimes as in Wyller *et al*. [15]. We have presented the results of the pattern forming process for four specific sets of the heterogeneity parameters $\alpha _{qp}$ listed in Table 2. These sets represent scenarios with weak, intermediate, and strong heterogeneity. We have divided the investigation into two subsections corresponding to the outcomes in the steep firing rate regime and in the shallow regime. Here we emphasize that the actual choices of steepness parameters and the heterogeneity parameters will of course not cover all possible outcomes, since all these parameters can be varied independently of each other.

The results of these simulations can be summarized as follows:

In the steep firing rate regime (Set *A*) we get as expected spatial oscillations. In the weakly modulated case (Set 1) the oscillations consist of periodically distributed bump-like structures where the shape of the solution restricted to one period overlaps remarkably well with the shape of the bumps detected in Kolodina *et al*. [33] for the firing rate functions modeled by means of the Heaviside function. This gives rise to the conjecture that the final outcome of the pattern formation process in the steep firing rate regime is 1-bump periodic solutions. The wavelength of these oscillations is as expected approximately equal to the wave length of the linearly most unstable mode. In the process of running numerical simulations, we have observed that it takes a relatively longer time to form the stabilized stationary patterns than in the translational invariant case. This is indeed expected since the growth rate of the linearly most unstable mode in this case is less than the corresponding growth rate in the translational invariant case. Beyond the weakly modulated case the numerical examples indicate a further slowdown of the pattern forming process for Set 2 and Set 3. Interestingly, the maximal growth rate produced when using Set 3 as input parameters is greater than the maximal growth rate for Set 1, see Fig. 1. In Set 4 the maximal growth rate is the smallest one, but the patterns form faster than in the case with Set 1 as input parameters. This shows that the parametric complexity of the present model can produce a rich plethora of phenomena, in particular in the regime beyond the weakly modulated case.

In the shallow firing rate regime (Set *B*) we obtain spatiotemporal oscillations. In the weakly modulated case (Set 1) spatiotemporal oscillations appear as homotopic deformation of the spatiotemporal oscillations of the translational invariant case. This is consistent with the fact that the stability matrix for the linearly most unstable mode are complex conjugate eigenvalues. In this case the increase in the heterogeneity parameter decreases the growth rate of the fastest growing mode, thus explaining the slowdown of the pattern forming process when increasing the heterogeneity parameter. We have also observed that the pattern forming process in the shallow firing rate regime takes a longer time than in the steep regime. This result can easily be understood as a consequence of the magnitude of order difference between the maximal growth rate in the steep firing rate regime versus the shallow firing rate regime. A numerical example shows that the relative inhibition time for the excitation of a gain band through a Turing–Hopf bifurcation increases with the heterogeneity parameter.

In the example with strong heterogeneity (Set 4), the effect of 1-periodicity in *y*-variable is visible in the numerical simulation results both in the steep firing rate regime (Set *A*) and in the shallow firing rate regime (Set *B*).

We conjecture that stable 1-bump periodic solutions (or their extension to *N*-bump periodic solutions) of homogenized model (4) can be constructed in a way analogous to Kolodina *et al*. [34]. It is a topic for future research to find out whether or not this type of solutions appears as the final outcome in a pattern forming process in the steep firing rate regime. In line with that it will be of interest to find out if this emerging structure can be approximated by means of periodically distributed stable single bumps of the type detected in Kolodina *et al*. [33]. This will shed light on the role of these single bumps. A natural extension of the present work consists of studying pattern formation within the modeling framework (4) when $N=2$. This means that a 2-population model with a periodic microstructure built into the connectivity kernels is defined on a two-dimensional domain. This extension can be viewed as a step towards a more realistic description of the actual situation in the cortical tissue. Other realistic effects which could be included in the present homogenized modeling framework (and its generalizations to Volterra type models) is finite axonal and dendritic delays effect. Here we will follow the line of thought as in Venkov *et al*. [43] and Faye *et al*. [44]. Finally, possible modifications of the present model consist of assuming other types of microstructure effects and then investigating existence and stability of coherent structures as well as pattern formation within the framework of the corresponding homogenized problems.

## Data Availability

Not applicable.

## Appendix A

The proof of Theorem 1 relies on the following bounding estimate for the nonlocal effects in the modeling framework.

### Lemma 1

*Let the firing rate functions* $P_{q}$ *and the connectivity kernels* $\omega _{qp}$ *be specified as in* (6)*–*(11) *and* (15). *Moreover*, *let* $\theta _{q}; q=e,i$ *be the threshold values in model* (3), $\Delta K_{qp}; q,p=e,i$ *the* $L^{1}$-*norm differences between* $\omega _{qp}$ *and the mean values* $\langle \omega _{qp}\rangle $ *defined by* (16), *and* $\|\cdot \|_{ {{\mathcal{B}}}}$ *the norm defined by* (12). *Then we have the estimate*

$$\begin{aligned} & \bigl\vert \omega _{qp}\otimes \otimes P_{q}(u_{q}- \theta _{q})-\langle \omega _{qp}\rangle \otimes P_{q}(\phi _{q}-\theta _{q}) \bigr\vert \\ &\quad\leq \Delta K_{qp}+\beta _{q}S_{\max }' \Vert u_{q}-\phi _{q} \Vert _{ {{\mathcal{B}}}}. \end{aligned}$$

### Proof

Straightforward estimation yields

$$\begin{aligned} & \bigl\vert \omega _{qp}\otimes \otimes P_{q}(u_{q}- \theta _{q})-\langle \omega _{qp}\rangle \otimes P_{q}(\phi _{q}-\theta _{q}) \bigr\vert \\ &\quad\leq \bigl\vert \bigl(\omega _{qp}-\langle \omega _{qp} \rangle \bigr)\otimes \otimes P_{q}(u_{q}- \theta _{q}) \bigr\vert + \bigl\vert \langle \omega _{qp} \rangle \otimes \otimes \bigl(P_{q}(u_{q}- \theta _{q})-P_{q}(\phi _{q}-\theta _{q}) \bigr) \bigr\vert \\ &\quad\leq \sup_{(x,y)\in \mathbb{R}\times \mathbb{T}}\biggl\{ \int _{\mathbb{R}} \biggl( \int _{\mathbb{T}} \bigl\vert \omega _{qp} \bigl(x'-x,y'-y\bigr)- \langle \omega _{qp} \rangle \bigl(x'-x\bigr) \bigr\vert \,dy' \biggr) \,dx'\biggr\} \\ &\qquad{}+\sup_{x\in \mathbb{R}}\biggl\{ \int _{\mathbb{R}} \langle \omega _{qp}\rangle \bigl(x'-x\bigr)\,dx'\biggr\} \beta _{q}S_{\max }' \Vert u_{q}- \phi _{q} \Vert _{ {{\mathcal{B}}}} \\ &\quad= \int _{\mathbb{R}}\biggl\{ \int _{\mathbb{T}} \bigl\vert \omega _{qp} \bigl(x',y'\bigr)- \langle \omega _{qp}\rangle \bigl(x'\bigr) \bigr\vert \,dy'\biggr\} \,dx'+\biggl\{ \int _{ \mathbb{R}}\langle \omega _{qp}\rangle \bigl(x'\bigr)\,dx'\biggr\} \beta _{q}S_{\max }' \Vert u_{q}-\phi _{q} \Vert _{ {{\mathcal{B}}}} \\ &\quad= \int _{\mathbb{R}}\biggl\{ \int _{\mathbb{T}} \bigl\vert \omega _{qp} \bigl(x',y'\bigr)- \langle \omega _{qp}\rangle \bigl(x'\bigr) \bigr\vert \,dy'\biggr\} \,dx'+\beta _{q}S_{\max }' \Vert u_{q}- \phi _{q} \Vert _{ {{\mathcal{B}}}} \\ &\quad=\Delta K_{qp}+\beta _{q}S_{\max }' \Vert u_{q}-\phi _{q} \Vert _{ {{\mathcal{B}}}} \end{aligned}$$

by exploiting the suspension trick $a-b=a-c+c-b$, the triangle inequality and the conditions imposed on the firing rate functions and the connectivity kernels. □

We are now in a position to prove Theorem 1.

### Proof

Introduce the temporal kernels

$$\begin{aligned} \alpha _{1}(t)=\exp [-t],\qquad \alpha _{\tau }(t)=\frac{1}{\tau } \exp [-t/ \tau ]. \end{aligned}$$

Let $U_{\langle \rangle }=(\phi _{e},\phi _{i})$ denote the solution of (1) with the mean values $\langle \omega _{qp}\rangle $ of the connectivity kernels $\omega _{qp}$ as connectivity kernels. We get the fixed point problems

A.1 $$\begin{aligned} \begin{aligned} \phi _{e}(x,T)={}&\phi _{e}(x,0)\alpha _{1}(T)\\ &{}+ \int _{0}^{T}\alpha _{1}(T-t) \bigl( \langle \omega _{ee} \rangle \otimes P_{e}(\phi _{e}-\theta _{e})- \langle \omega _{ie} \rangle \otimes P_{i}(\phi _{i}-\theta _{i}) \bigr) (x,t) \,dt,\\ \phi _{i}(x,T)={}&\phi _{i}(x,0)\tau \alpha _{\tau }(T)\\ &{}+ \int _{0}^{T}\alpha _{\tau }(T-t) \bigl( \langle \omega _{ei} \rangle \otimes P_{e}(\phi _{e}-\theta _{e})- \langle \omega _{ii} \rangle \otimes P_{i}(\phi _{i}-\theta _{i}) \bigr) (x,t) \,dt \end{aligned} \end{aligned}$$

*t* from 0 to *T*. Formal integration of (4) with respect to time *t* from 0 to *T* yields the fixed point problems

A.2 $$\begin{aligned} \begin{aligned} u_{e}(x,y,T)={}&u_{e}(x,y,0)\alpha _{1}(T)\\ &{} + \int _{0}^{T}\alpha _{1}(T-t) \bigl( \omega _{ee}\otimes \otimes P_{e}(u_{e}-\theta _{e})- \omega _{ie}\otimes \otimes P_{i}(u_{i}- \theta _{i}) \bigr) (x,y,t)\,dt, \\ u_{i}(x,y,T)={}&u_{i}(x,y,0)\tau \alpha _{\tau }(T)\\ &{}+ \int _{0}^{T}\alpha _{\tau }(T-t) \bigl( \omega _{ei}\otimes \otimes P_{e}(u_{e}-\theta _{e})- \omega _{ii}\otimes \otimes P_{i}( \phi _{i}-\theta _{i}) \bigr) (x,y,t)\,dt \end{aligned} \end{aligned}$$

for the component functions $u_{e}$ and $u_{i}$ of the solution $U=(u_{e},u_{i})$. We then get the inequalities

A.3 $$\begin{aligned} \begin{aligned} & \vert u_{e}-\phi _{e} \vert (x,y,T)\\ &\quad\leq \vert u_{e}-\phi _{e} \vert (x,y,0) \\ & \qquad{}+ \int _{0}^{T} \bigl\vert \bigl(\omega _{ee}\otimes \otimes P_{e}(u_{e}- \theta _{e})-\langle \omega _{ee}\rangle \otimes P_{e}( \phi _{e}- \theta _{e}) \bigr) (x,y,t) \bigr\vert \,dt \\ &\qquad{}+ \int _{0}^{T} \bigl\vert \bigl(\omega _{ie}\otimes \otimes P_{i}(u_{i}- \theta _{i})-\langle \omega _{ie}\rangle \otimes P_{i}( \phi _{i}- \theta _{i}) \bigr) (x,y,t) \bigr\vert \,dt, \\ &\vert u_{i}-\phi _{i} \vert (x,y,T)\\ &\quad\leq \vert u_{i}-\phi _{i} \vert (x,y,0) \\ &\qquad{}+\frac{1}{\tau } \biggl[ \int _{0}^{T} \bigl\vert \bigl(\omega _{ei}\otimes \otimes P_{e}(u_{e}-\theta _{e})-\langle \omega _{ei}\rangle \otimes P_{e}( \phi _{e}-\theta _{e}) \bigr) (x,y,t) \bigr\vert \,dt \\ &\qquad{}+ \int _{0}^{T} \bigl\vert \bigl(\omega _{ii}\otimes \otimes P_{i}(u_{i}- \theta _{i})-\langle \omega _{ii}\rangle \otimes P_{i}( \phi _{i}- \theta _{i}) \bigr) (x,y,t) \bigr\vert \,dt \biggr] \end{aligned} \end{aligned}$$

by using (A.1) and (A.2). In the process of deriving these inequalities, we have also made use of the fact that

$$\begin{aligned} 0< \alpha _{1}(t)\leq 1,\quad 0< \tau \alpha _{\tau }(t)\leq 1. \end{aligned}$$

By combining Lemma 1, (16), and (A.3), we readily find that

$$\begin{aligned} & \Vert u_{e}-\phi _{e} \Vert _{ {{\mathcal{B}}}}(T)\\ &\quad\leq \Vert u_{e}- \phi _{e} \Vert _{ {{\mathcal{B}}}}(0)+(\Delta K_{ee}+ \Delta K_{ie})T+\beta _{e}S_{\mathrm{max}}' \int _{0}^{T} \Vert u_{e}-\phi _{e} \Vert _{ {{\mathcal{B}}}}(t)\,dt\\ &\qquad{}+\beta _{i}S_{\mathrm{max}}' \int _{0}^{T} \Vert u_{i}-\phi _{i} \Vert _{ {{\mathcal{B}}}}(t)\,dt, \\ & \Vert u_{i}-\phi _{i} \Vert _{ {{\mathcal{B}}}}(T)\\ &\quad \leq \Vert u_{i}- \phi _{i} \Vert _{ {{\mathcal{B}}}}(0)+ \frac{1}{\tau } \biggl[( \Delta K_{ei}+\Delta K_{ii})T +\beta _{e}S_{\mathrm{max}}' \int _{0}^{T} \Vert u_{e}-\phi _{e} \Vert _{ {{\mathcal{B}}}}(t)\,dt\\ &\qquad{}+\beta _{i}S_{\mathrm{max}}' \int _{0}^{T} \Vert u_{i}-\phi _{i} \Vert _{ {{\mathcal{B}}}}(t)\,dt \biggr], \end{aligned}$$

from which it follows that

$$\begin{aligned} D(T)\leq {}&D(0)+ \biggl[\Delta K_{ee}+\Delta K_{ie}+ \frac{1}{\tau }( \Delta K_{ei}+\Delta K_{ii}) \biggr]T \\ &{}+\biggl(1+\frac{1}{\tau }\biggr)\beta _{\max }S_{\max }' \int _{0}^{T}D(t)\,dt, \end{aligned}$$

where $\beta _{\max }\equiv \max (\beta _{e},\beta _{i})$. Here we have made use of definition (17) of the separation distance *D* between the solutions $U=(u_{e},u_{i})$ and $U_{\langle \rangle }=(\phi _{e},\phi _{i})$. Noticing that *D* is a continuous function of *t* on the interval $[0,+\infty )$ and the linear function *M* defined by (19) is nondecreasing, we end up with inequality (18) by appealing to the integral form of Grönwall’s lemma [45]. □

## Appendix B

Here we show that the gain band structure is of the finite bandwidth type when the scaling function Φ of the connectivity kernels $\omega _{qp}$ defined by (8) is satisfying the localization property (10) in addition to (9). This conclusion is based on the following theorems and corollary.

### Theorem 2

*The Fourier coefficients* $\hat{\omega }_{qp}^{(n)}$, $n=0,1,2,\ldots $ , *defined by* (36) *are continuous functions of* *k* *satisfying the bounding inequality*

B.1 $$\begin{aligned} \hat{\omega }_{qp}^{(n)}(k)\rightarrow 0 \quad\textit{as } \vert k \vert \rightarrow \infty \textit{ for all $n\in \mathbb{N}_{0}$}. \end{aligned}$$

### Proof

According to (9), the scaling function Φ of the connectivity kernels $\omega _{qp}$ defined by (8) is absolute integrable. This implies that the Fourier transform Φ̃ of Φ is a uniformly continuous function of *k*. See, for example, Kolmogorov *et al*. [37] for further details. Hence, by (34), the composite mapping $\tilde{\Phi }\circ S_{qp}^{(k)}:\mathbb{T}\rightarrow \mathbb{R}$ defined by

B.2 $$\begin{aligned} y\overset{S_{qp}^{(k)}}{\longmapsto } k\sigma _{qp}(y) \overset{\tilde{\Phi }}{\longmapsto }\tilde{\Phi } \bigl(k \sigma _{qp}(y) \bigr)=\tilde{\omega }_{qp}(k,y) \end{aligned}$$

is a continuous function of $y\in \mathbb{T}$ for all $k\in \mathbb{R}$. Here $S_{qp}^{(k)}(y)\equiv k\sigma _{qp}(y)$. Hence, Fourier coefficients $\{\hat{\omega }_{qp}^{(n)}\}_{n=0}^{\infty }$ are continuous functions of *k*. Moreover, we find according to Riemann–Lebesque’s lemma that $\tilde{\Phi }(k)\rightarrow 0$ as $|k|\rightarrow \infty $. By (34), we find that $\tilde{\omega }_{qp}(k,y)\rightarrow 0$ as $|k|\rightarrow \infty $ uniformly in $y\in \mathbb{T}$ from which (B.1) follows. □

### Theorem 3

*Assume that the scaling function* Φ *satisfies the localization property* (10) *for some natural number* $r\geq 1$. *Then the Fourier coefficients* $\hat{\omega }_{qp}^{(n)}$, $n=0,1,2,\ldots $ , *defined by* (36) *are* *r* *times continuously differentiable functions of k and satisfy the bounds*

B.3 $$\begin{aligned} \bigl\vert \hat{\omega }_{qp}^{(n)}(k) \bigr\vert \leq \frac{A_{qp}(k)}{n^{r}},\quad k\in \mathbb{R}, \end{aligned}$$

*where* $A_{qp}(k)$ *are strictly positive constants which are independent of* *n*.

### Proof

Let Φ̃ denote the Fourier transform of Φ. The localization property (10) implies that Φ̃, $\frac{d}{dk}\tilde{\Phi }$,…,$\frac{d^{r}}{dk^{r}}\tilde{\Phi }$ are uniformly continuous functions of *k*. Here we again make use of general results for Fourier transforms, see Kolmogorov *et al*. [37]. Again, we study the composite function $\tilde{\Phi }\circ S_{qp}^{(k)}:\mathbb{T}\rightarrow \mathbb{R}$ defined by (B.2). We readily conclude that the functions $\tilde{\Phi }\circ S_{qp}^{(k)}$, $\frac{\partial }{\partial k}(\tilde{\Phi }\circ S_{qp}^{(k)})$,…,$\frac{\partial ^{r}}{\partial k^{r}}(\tilde{\Phi }\circ S_{qp}^{(k)})$ are uniformly continuous functions of $y\in \mathbb{T}$ for all $k\in \mathbb{R}$ from which it follows that the Fourier coefficients $\{\hat{\omega }_{qp}^{(n)}\}_{n=0}^{\infty }$ are *r* times continuously differentiable with respect to *k* with

$$\begin{aligned} \frac{d^{l}}{dk^{l}}\hat{\omega }_{qp}^{(n)}(k)= \int _{ \mathbb{T}}\frac{\partial ^{l}}{\partial k^{l}} \bigl(\tilde{\Phi }\bigl(k \sigma _{qp}(y)\bigr) \bigr)\exp [-i2\pi n y]\,dy,\quad l=0,1,\ldots,r. \end{aligned}$$

Next, since $S_{qp}^{(k)}(y)\equiv k\sigma _{qp}(y)$, $\frac{d}{dy}S_{qp}^{(k)}$,…,$\frac{d^{r}}{dy^{r}}S_{qp}^{(k)}$ are continuous and 1-periodic for all k, the functions $\tilde{\Phi }\circ S_{qp}^{(k)}$, $\frac{d}{dy}(\tilde{\Phi }\circ S_{qp}^{(k)})$,…,$\frac{d^{r}}{dy^{r}}(\tilde{\Phi }\circ S_{qp}^{(k)})$ are also continuous and 1-periodic in *y* for each k. Therefore, by successive partial integrations and the bounding inequality $|\int _{\mathbb{T}}g(y)\,dy|\leq \max_{y\in \mathbb{T}}|g(y)|$ for any continuous function $g:\mathbb{T}\rightarrow \mathbb{C}$, we find that the Fourier coefficients $\hat{\omega }_{qp}^{(n)}$, $n\in \mathbb{N}_{0}$ defined by (36) satisfy the bounds (B.3) with

$$\begin{aligned} A_{qp}(k)=\frac{1}{(2\pi )^{r}}\max_{y\in \mathbb{T}} \biggl\vert \frac{d^{r}}{dy^{r}} \bigl(\tilde{\Phi }\bigl(k\sigma _{qp}(y)\bigr) \bigr) \biggr\vert . \end{aligned}$$

 □

We immediately get the following corollary from Theorem 3.

### Corollary 1

*If* (10) *is satisfied for* $r\geq 1$, *then the Fourier coefficients* $\hat{\omega }_{qp}^{(n}$, $n=0,1,2,\ldots $ , *are smooth functions of* *k* *and satisfy the*

$$\begin{aligned} \hat{\omega }_{qp}^{(n)}(k)\rightarrow 0 \quad\textit{as } n \rightarrow \infty \textit{ uniformly in $k$}. \end{aligned}$$

Next, let us investigate the role of the stability matrix $\mathbf{A}_{0}$ defined by means of (40) (with $n=0$).

### Theorem 4

*Let* $\langle \omega _{qp}\rangle $ *denote the mean value of the connectivity kernel* $\omega _{qp}$ *defined by means of* (15). *Then the stability matrix emerging in the linear stability analysis of the equilibrium* $U_{0}=(v_{0},v_{0})$ *is given by means of matrix* (42).

### Proof

This result follows directly from the computation of the Fourier transform $\widetilde{\langle \omega _{qp}\rangle }$ of the mean value of $\omega _{qp}$:

$$\begin{aligned} \widetilde{\langle \omega _{qp}\rangle }(k)&= \int _{ \mathbb{R}}\langle \omega _{qp}\rangle (x)\exp (-i2\pi xk)\,dx \\ &= \int _{\mathbb{R}} \biggl( \int _{\mathbb{T}}\omega _{qp}(x,y)\,dy \biggr)\exp (-i2\pi xk)\,dx\\ &= \int _{\mathbb{T}} \biggl( \int _{ \mathbb{R}}\,dx\omega _{qp}(x,y)\exp (-i2\pi xk)\,dx \biggr)\,dy\\ &= \int _{\mathbb{T}}\widetilde{\omega }_{qp}(k,y)\,dy=\langle \tilde{\omega }_{mn}\rangle (k)=\hat{\omega }_{qp}^{(0)}(k). \end{aligned}$$

Here we have made use of Fubini’s theorem for the interchange of the order of integration. □

The conclusion regarding the gain band structure is based on the following theorem.

### Theorem 5

*Assume that the scaling function* Φ *satisfies the localization property* (10) *for some* $r\geq 1$. *Then the functions* $\varphi _{n}$ *or* $\psi _{n}$ *given as* (44) *are smooth functions of* *η*. *Moreover*, *the number of transversal crossings of the curves* $\Gamma _{n}=(\varphi _{n},\psi _{n})$, $n=1,2,\ldots,n_{0}$, *with the positive* $\psi _{n}$-*axis and the negative* $\varphi _{n}$-*axis is finite*.

### Proof

By Corollary 1 and definition (44), the trace and determinant functions $\varphi _{n}$ and $\psi _{n}$ are smooth functions of *η*. The transversal crossings of $\Gamma _{n}=(\varphi _{n},\psi _{n})$, $n=1,2,\ldots,n_{0}$, with the positive $\psi _{n}$-axis and the negative $\varphi _{n}$-axis take place for values *η* satisfying

B.4 $$\begin{aligned} \varphi _{n}(\eta )=0,\qquad \frac{\partial }{\partial \eta }\varphi _{n}( \eta )\neq 0,\quad \psi _{n}(\eta )>0 \end{aligned}$$

and

B.5 $$\begin{aligned} \psi _{n}(\eta )=0,\qquad \frac{\partial }{\partial \eta }\psi _{n}( \eta ) \neq 0,\quad \varphi _{n}(\eta )< 0. \end{aligned}$$

We first prove that the zeros of $\varphi _{n}$ or $\psi _{n}$ are isolated and distinct by means of a contradiction argument. Let $\{\eta _{m}\}_{m=1}^{\infty }$ be a convergent sequence of zeros of $f_{n}=\varphi _{n},\psi _{n}$, i.e., that $\lim_{m\rightarrow \infty }\eta _{m}=\eta ^{\ast }$. Then, by the continuity of $f_{n}=\varphi _{n},\psi _{n}$, we find that

$$\begin{aligned} 0=\lim_{m\rightarrow \infty }f_{n}(\eta _{m})=f_{n} \Bigl(\lim_{m\rightarrow \infty }\eta _{m}\Bigr)=f_{n} \bigl(\eta ^{\ast }\bigr), \end{aligned}$$

which means that the accumulation point $\eta ^{\ast }$ is a zero of $f_{n}$. Thus any open interval about $\eta ^{\ast }$ contains at least one zero $\eta _{m}$. But the transversality condition $\frac{\partial }{\partial \eta }f_{n}(\eta ^{\ast })\neq 0$ implies that there is an open interval $I= (\eta ^{\ast }-\Delta \eta,\eta ^{\ast } +\Delta \eta )$ such that $f_{n}(\eta )\neq 0$ for $\eta \in I$ and $\eta \neq \eta ^{\ast }$. Therefore no zero of $f_{n}$ can be an accumulation point of some sequence of zeros of $f_{n}$. Hence the zeros of $f_{n}$ must be isolated and distinct. Next, let us prove that the number of zeros of $f_{n}$ must be finite. The continuity of $f_{n}$ and the fact that the curve $\Gamma _{n}$ terminates in the second quadrant in the $(\varphi _{n},\psi _{n})$-plane imply that there is a zero denoted by $\eta _{\max }$ of either $\varphi _{n}$ or $\psi _{n}$ such that the curve $\Gamma _{n}$ remains in the second quadrant for $\eta >\eta _{\max }$. This means that all the zeros of $f_{n}=\varphi _{n},\Psi _{n}$ satisfying (B.4) and (B.5) are located in the half-open interval $(0,\eta _{\max }]$. This result together with the fact that the zeros are isolated and distinct leads to the conclusion that the number of zeros is finite. Hence we can only have a finite number of transversal crossings of $\Gamma _{n}$ of types (B.4) and (B.5). □

### Remark 1

The proof of Theorem 5 proceeds in a way analogous to the proof of Theorem 1 in the appendix in Wyller *et al*. [41]. For the sake of completeness, we have included this theorem and its corresponding proof in the present paper.

We notice that the exponentially decaying function Φ defined by (21) satisfies the localization property (10) for all $r\in \mathbb{N}_{0}$. The numerical findings in Sect. 4 are indeed consistent with both Theorem 1, Theorem 2, Theorem 3, and Theorem 5.

## Appendix C

Let us assemble three of the heterogeneity parameters into a single parameter vector which we denote by *â*. The remaining heterogeneity parameter is denoted by *α̂*. We fix *a*. The following result serves as a basis for the Turing–Hopf theory developed in Section.

### Theorem 6

*Assume that condition* (10) *is fulfilled for* $r\geq 2$. *Assume that system* (58) *is fulfilled for* $n=0$ *and* $\hat{\alpha }=0$, *i*.*e*., *that there is a point* $(\eta _{c,0},\tau _{c,0})$ *such that*

C.1 $$\begin{aligned} \begin{aligned} &\varphi _{0}(\eta _{c,0},\tau _{c,0},0)=0,\qquad \frac{\partial \varphi _{0}}{\partial \eta }(\eta _{c,0},\tau _{c,0},0)=0, \\ &\psi _{0}(\eta _{c,0},\tau _{c,0},0)>0,\qquad \frac{\partial ^{2}\varphi _{0}}{\partial \eta ^{2}}(\eta _{c,0}, \tau _{c,0},0)\neq 0,\qquad \frac{\partial \varphi _{0}}{\partial \tau }(\eta _{c,0},\tau _{c,0},0) \neq 0. \end{aligned} \end{aligned}$$

*Then there is a unique and smooth solution* $(\eta,\tau )=(\eta _{c}(\hat{\alpha }),\tau _{c}(\hat{\alpha }))$ *for* $\hat{\alpha }\in [0,\varepsilon )$ (*with* $0\leq \varepsilon < 1$) *of system* (58) *for which the conditions*

C.2 $$\begin{aligned} \psi _{0}\bigl(\eta _{c}(\hat{\alpha }),\tau _{c}(\hat{\alpha }); \hat{\alpha }\bigr)>0,\qquad \frac{\partial ^{2}}{\partial \eta ^{2}} \varphi _{0}\bigl(\eta _{c}(\hat{\alpha }),\tau _{c}( \hat{\alpha }); \hat{\alpha }\bigr)\neq 0 \end{aligned}$$

*are fulfilled for* *α̂* *in some interval about* $\hat{\alpha }=0$.

### Proof

According to Theorem 3 in Appendix B, condition (10) for $r\geq 2$ implies that the function $\varphi _{0}$ defined by means of (44) is at least two times continuously differentiable with respect to *η*. Let Σ and *I* be the set

$$\begin{aligned} \Sigma =\bigl\{ (\eta,\tau )\in \mathbb{R}^{2}; \eta \geq 0,\tau >0 \bigr\} , I=[0,1) \end{aligned}$$

and introduce the smooth vector-valued function $G_{0}:\Sigma \times I\rightarrow \mathbb{R}^{2}$ defined by

$$G_{0}(\eta,\tau,\hat{\alpha })\equiv \begin{bmatrix} \varphi _{0}(\eta,\tau,\hat{\alpha }) \\ \frac{\partial \varphi _{0}}{\partial \eta }(\eta,\tau,\hat{\alpha }) \end{bmatrix}, $$

where $(\eta,\tau )\in \Omega, \hat{\alpha }\in I$. According to the assumption $(\eta _{c,0},\tau _{c,0},0)$ is a solution of the system $G_{0}(\eta,\tau,\hat{\alpha })=0$ when $\hat{\alpha }=0$. We readily find that the Jacobian $D_{(\eta,\tau )}G_{0}$ of $G_{0}$ evaluated at the point $(\eta _{c,0},\tau _{c,0},0)$ is given as

$$\begin{aligned} D_{(\eta,\tau )}G_{0}(\eta _{c,0},\tau _{c,0},0)= \begin{pmatrix} 0&\frac{\partial \varphi _{0}}{\partial \tau }(\eta _{c,0},\tau _{c,0},0) \\ \frac{\partial ^{2}\varphi _{0}}{\partial \eta ^{2}}(\eta _{c,0}, \tau _{c,0},0)&\frac{\partial ^{2}\varphi _{0}}{\partial \eta \partial \tau }( \eta _{c,0},\tau _{c,0},0) \end{pmatrix}, \end{aligned}$$

from which it follows that

$$\begin{aligned} \det \bigl\{ D_{(\eta,\tau )}G_{0}(\eta _{c,0},\tau _{c,0},0)\bigr\} =- \frac{\partial \varphi _{0}}{\partial \tau }(\eta _{c,0},\tau _{c,0},0) \cdot \frac{\partial ^{2}\varphi _{0}}{\partial \eta ^{2}}(\eta _{c,0}, \tau _{c,0},0). \end{aligned}$$

Assumption (C.2) implies that

$$\begin{aligned} \det \bigl\{ D_{(\eta,\tau )}G_{0}(\eta _{c,0},\tau _{c,0},0)\bigr\} \neq 0, \end{aligned}$$

from which it follows that $D_{(\eta,\tau )}G_{0}(\eta _{c,0},\tau _{c,0},0)$ is non-singular. The implicit function theorem then implies that there is a unique smooth parametrization $\mathcal{C}: (\eta,\tau )=(\eta _{c}(\hat{\alpha }),\tau _{c}( \hat{\alpha }))$, where $\hat{\alpha }\in [0,\varepsilon )$ for some *ε* such that $0\leq \varepsilon <1$ for which $G_{0}(\eta _{c}(\hat{\alpha }),\tau _{c}(\hat{\alpha }),\hat{\alpha })=0$. The continuity of the functions $\psi _{0}$ and $\frac{\partial ^{2}}{\partial \eta ^{2}}\varphi _{0}$ implies that conditions (C.2) are fulfilled for some open interval about $\hat{\alpha }=0$. □

### Example

The exponentially decaying scaling function Φ defined by (21) satisfies condition (10) for all $r\geq 0$. Assume that $a=0$. According to Wyller *et al*. [15], system (C.1) has a unique solution for the relative inhibition time $\tau _{c,0}$ satisfying the bound

$$\begin{aligned} \tau _{\ast }< \tau _{c,0}< \tau _{H} \end{aligned}$$

when $P_{e}'>1$ and $s_{ii}>s_{ee}$. Here $\tau _{H}$ is defined by (24) and $\tau _{\ast }$ by

$$\begin{aligned} \tau _{\ast }\equiv \frac{P_{i}'+1+\frac{s_{ii}^{2}}{s_{ee}^{2}}}{(P_{e}'-1)\frac{s_{ii}^{2}}{s_{ee}^{2}}-1}. \end{aligned}$$

Let $\eta _{c,0}=k_{c,0}^{2}$. We readily find that

$$\begin{aligned} \frac{\partial \varphi _{0}}{\partial \tau }(\eta _{c,0},\tau _{c,0},0)=1+P_{i}' \tilde{\Phi }_{ii}(s_{ii}k_{c,0})=1+P_{i}' \frac{1}{4\pi ^{2}s_{ii}^{2}\eta _{c,0}+1}>0 \end{aligned}$$

and

$$\begin{aligned} \frac{\partial ^{2}\varphi _{0}}{\partial \eta ^{2}}(\eta _{c,0}, \tau _{c,0},0)=- \frac{32\pi ^{4}(1+\tau _{c,0}^{-1})s_{ee}^{2}s_{ii}^{2}}{(4\pi ^{2}s_{ii}^{2}\eta _{c,0}+1)(4\pi ^{2}s_{ee}^{2}\eta _{c,0}+1)}< 0. \end{aligned}$$

Moreover, we get

$$\begin{aligned} \psi _{0}(\eta _{c,0},\tau _{c,0},0)=\tau ^{-1} \frac{H_{4}(\eta _{c,0})}{(1+4\pi ^{2}s_{ee}^{2}\eta )(1+4\pi ^{2}s_{ii}^{2}\eta _{c,0})(1+4\pi ^{2}s_{ei}^{2}\eta _{c,0})(1+4\pi ^{2}s_{ie}^{2}\eta _{c,0})}, \end{aligned}$$

where $H_{4}$ is a quartic polynomial in *η* derived in [15]. Due to the complexity of the analytical expression for $H_{4}$, it is not possible to infer simple analytical predictions regarding the sign of $\psi _{0}$. We thus rely on numerical computations to resolve this issue. Notice, however, that for the numerical example demonstrated in Sect. 4, it is shown in [15] that $H_{4}(\eta _{c,0})>0$. Hence $\psi _{0}(\eta _{c,0},\tau _{c,0},0)>0$ in the present example. Theorem 6 then implies that there is a unique smooth solution $(\eta,\tau )=(\eta _{c}(\hat{\alpha }),\tau _{c}(\hat{\alpha }))$ of system (C.1) with $\eta _{c}(0)=\eta _{c,0}$ and $\tau _{c}(0)=\tau _{c,0}$ for which condition (C.2) is satisfied in the weakly modulated case ($0<\hat{\alpha }\ll 1$). Moreover, since $\frac{\partial ^{2}\varphi _{0}}{\partial \eta ^{2}}(\eta _{c,0}, \tau _{c,0},0)<0$, an excitation of a gain band is expected to take place in the weakly modulated case ($0<\hat{\alpha }\ll 1$). This analytically based prediction is supported by the findings summarized in Fig. 6 in Sect. 4.

## References

[1] Amari S-I (1975). Homogeneous nets of neuron-like elements. Biol Cybern.

[2] Amari S (1977). Dynamics of pattern formation in lateral-inhibition type neural fields. Biol Cybern.

[3] Wilson HR, Cowan JD (1972). Excitatory and inhibitory interactions in localized populations of model neurons. Biophys J.

[4] Wilson HR, Cowan JD (1973). A mathematical theory of the functional dynamics of cortical and thalamic nervous tissue. Kybernetik.

[5] Bressloff PC (2011). Spatiotemporal dynamics of continuum neural fields. J Phys A, Math Theor.

[6] Bressloff PC, Cowan JD, Golubitsky M, Thomas PJ (2001). Scalar and pseudoscalar bifurcations motivated by pattern formation on the visual cortex. Nonlinearity.

[7] Bressloff PC, Cowan JD, Golubitsky M, Thomas PJ, Wiener MC (2001). Geometric visual hallucinations, Euclidean symmetry and the functional architecture of striate cortex. Philos Trans R Soc Lond B, Biol Sci.

[8] Ermentrout GB, Cowan JD (1979). A mathematical theory of visual hallucination patterns. Biol Cybern.

[9] Goldman-Rakic PS (1995). Cellular basis of working memory. Neuron.

[10] Laing CR, Troy WC, Gutkin B, Bard Ermentrout G (2002). Multiple bumps in a neuronal model of working memory. SIAM J Appl Math.

[11] Laing CR, Troy WC (2003). Two-bump solutions of Amari-type models of working memory. Phys D, Nonlinear Phenom.

[12] Ermentrout GB, McLeod JB (1993). Existence and uniqueness of travelling waves for a neural network. Proc R Soc Edinb, Sect A, Math.

[13] Huang X, Troy WC, Yang Q, Ma H, Laing CR, Schiff SJ, Wu J-Y (2004). Spiral waves in disinhibited mammalian neocortex. J Neurosci.

[14] Blomquist P, Wyller J, Einevoll GT (2005). Localized activity patterns in two-population neuronal networks. Phys D, Nonlinear Phenom.

[15] Wyller J, Blomquist P, Einevoll GT (2007). Turing instability and pattern formation in a two-population neuronal network model. Phys D, Nonlinear Phenom.

[16] Xin J (2000). An introduction to fronts in random media. SIAM Rev.

[17] Xin J (2009). An introduction to fronts in random media.

[18] Bressloff PC (2001). Traveling fronts and wave propagation failure in an inhomogeneous neural network. Phys D, Nonlinear Phenom.

[19] Bressloff PC (2003). Spatially periodic modulation of cortical patterns by long-range horizontal connections. Phys D, Nonlinear Phenom.

[20] Bressloff PC, Folias SE, Prat A, Li Y-X (2003). Oscillatory waves in inhomogeneous neural media. Phys Rev Lett.

[21] Kilpatrick ZP, Folias SE, Bressloff PC (2008). Traveling pulses and wave propagation failure in inhomogeneous neural media. SIAM J Appl Dyn Syst.

[22] Coombes S, Laing CR (2011). Pulsating fronts in periodically modulated neural field models. Phys Rev E.

[23] Schmidt H, Hutt A, Schimansky-Geier L (2009). Wave fronts in inhomogeneous neural field models. Phys D, Nonlinear Phenom.

[24] Coombes S, Laing C, Schmidt H, Svanstedt N, Wyller J (2012). Waves in random neural media. Discrete Contin Dyn Syst, Ser A.

[25] Svanstedt N, Woukeng JL (2013). Homogenization of a Wilson–Cowan model for neural fields. Nonlinear Anal, Real World Appl.

[26] Svanstedt N, Wyller J, Malyutina E (2014). A one-population Amari model with periodic microstructure. Nonlinearity.

[27] Nguetseng G (1989). A general convergence result for a functional related to the theory of homogenization. SIAM J Math Anal.

[28] Lukkassen D, Nguetseng G, Wall P (2002). Two-scale convergence. Int J Pure Appl Math.

[29] Visintin A (2006). Towards a two-scale calculus. ESAIM Control Optim Calc Var.

[30] Malyutina E, Wyller J, Ponosov A (2014). Two bump solutions of a homogenized Wilson–Cowan model with periodic microstructure. Phys D, Nonlinear Phenom.

[31] Malyutina E, Ponosov A, Wyller J (2015). Numerical analysis of bump solutions for neural field equations with periodic microstructure. Appl Math Comput.

[32] Burlakov E, Wyller J, Ponosov A (2016). Two-dimensional Amari neural field model with periodic microstructure: rotationally symmetric bump solutions. Commun Nonlinear Sci Numer Simul.

[33] Kolodina K, Oleynik A, Wyller J (2018). Single bumps in a 2-population homogenized neuronal network model. Phys D, Nonlinear Phenom.

[34] Kolodina K, Kostrykin V, Oleynik A. Existence and stability of periodic solutions in a neural field equation. 2017. arXiv preprint. arXiv:1712.09688.

[35] Potthast R, Beim Graben P (2010). Existence and properties of solutions for neural field equations. Math Methods Appl Sci.

[36] Burlakov E, Zhukovskiy E, Ponosov A, Wyller J (2015). Existence, uniqueness and continuous dependence on parameters of solutions to neural field equations.

[37] Kolmogorov AN, Fomin SV. Elements of the theory of functions and functional analysis. vol. 1. Courier Corporation; 1957.

[38] Burlakov E, Ponosov A, Wyller J (2016). Stationary solutions of continuous and discontinuous neural field equations. J Math Anal Appl.

[39] Oleynik A, Ponosov A, Wyller J (2013). On the properties of nonlinear nonlocal operators arising in neural field models. J Math Anal Appl.

[40] Oleynik A, Ponosov A, Kostrykin V, Sobolev AV (2016). Spatially localized solutions of the Hammerstein equation with sigmoid type of nonlinearity. J Differ Equ.

[41] Wyller J, Krolikowski W, Bang O, Rasmussen JJ (2002). Generic features of modulational instability in nonlocal Kerr media. Phys Rev E.

[42] Wyller J, Królikowski WZ, Bang O, Petersen DE, Rasmussen JJ (2007). Modulational instability in the nonlocal $\chi ^{(2)}$-model. Phys D, Nonlinear Phenom.

[43] Venkov NA, Coombes S, Matthews PC (2007). Dynamic instabilities in scalar neural field equations with space-dependent delays. Phys D, Nonlinear Phenom.

[44] Faye G, Faugeras O (2010). Some theoretical and numerical results for delayed neural field equations. Phys D, Nonlinear Phenom.

[45] Bellman R (1943). Almost periodic gap series. Duke Math J.

